# Astragaloside II promotes intestinal epithelial repair by enhancing L-arginine uptake and activating the mTOR pathway

**DOI:** 10.1038/s41598-017-12435-y

**Published:** 2017-09-26

**Authors:** Shih-Yu Lee, Wei-Cheng Tsai, Jung-Chun Lin, Blerina Ahmetaj-Shala, Su-Feng Huang, Wen-Liang Chang, Tsu-Chung Chang

**Affiliations:** 1 Graduate Institute of Aerospace and Undersea Medicine, National Defense Medical Center, Taipei, Taiwan; 2 Department of Biochemistry, National Defense Medical Center, Taipei, Taiwan; 3 Division of Gastroenterology and Hepatology, Department of Internal Medicine, Tri-Service General Hospital, National Defense Medical Center, Taipei, Taiwan; 4 National Heart & Lung Institute, Imperial College London, London, United Kingdom; 5 School of Pharmacy, National Defense Medical Center, Taipei, Taiwan; 6 China Medical University Hospital, China Medical University, Taichung, Taiwan; 7 Department of Biotechnology, Asia University, Taichung, Taiwan

## Abstract

Astragaloside II (AS II) extracted from *Astragalus membranaceus* has been reported to promote tissue wound repair. However, the effect of AS II on inflammatory bowel disease is unknown. We investigated the effects and mechanism of AS II on intestinal wound healing in both *in vitro* and *in vivo* models. Human intestinal Caco-2 cells were treated with multiple concentrations of AS II to assess cell proliferation, scratch wound closure, L-arginine uptake, cationic amino acid transporter activity, and activation of the mTOR signaling pathway. These effects were also measured in a mouse model of colitis. AS II promoted wound closure and increased cell proliferation, L-arginine uptake, CAT1 and CAT2 protein levels, total protein synthesis, and phosphorylation of mTOR, S6K, and 4E-BP1 in Caco-2 cells. These effects were suppressed by lysine or rapamycin treatment, suggesting that the enhanced arginine uptake mediates AS II-induced wound healing. Similar results were also observed *in vivo*. Our findings indicate that AS II can contribute to epithelial barrier repair following intestinal injury, and may offer a therapeutic avenue in treating irritable bowel disease.

## Introduction

Inflammatory bowel disease (IBD) is a chronic gastrointestinal disorder, which can manifest as ulcerative colitis and Crohn’s disease^1^. Clinical symptoms include weight loss, abdominal pain, diarrhea, and bleeding^2^, and continuous mucosal inflammation can lead to intestinal fibrosis and may subsequently progress and develop into colon cancer^3^. The prevalence of IBD has been reported as 200 per 100,000 in the US, and this number is increasing; IBD has now become a global health issue as more countries are adopting a Western diet^4^. Although the precise mechanisms of IBD are still unknown, most studies concur that IBD is associated with hereditary, infectious, environmental, and auto-immune factors. The integrity of the intestinal epithelial barrier plays a role in IBD progression^5^. Recent studies have indicated that restoration of the epithelial barrier integrity is an important healing response in IBD and other intestinal disorders^6–10^. Thus, the repair of the intestinal epithelial barrier may be a promising therapeutic strategy in IBD. Current medications, such as non-steroidal anti-inflammatory drugs, steroids, and immunodulators, are limited in their application because of poor efficacy and adverse effects^10^. Therefore, a new effective therapy for IBD is needed.

Recovery of the epithelial barrier is crucial in the treatment of colitis. L-arginine (L-Arg) is involved in protein synthesis and regulation of many essential cellular functions, including immune response, hormone secretion, and wound healing^11^. In addition, L-Arg and its metabolite ornithine promote colonic epithelial wound repair by enhancing cell proliferation and collagen deposition^12^. L-Arg uptake has been shown to occur primarily by cationic amino acid transporter 2 (CAT 2), and is an important process in the restoration of colonic epithelial cells^10^. This is also confirmed by evidence that L-Arg supplementation suppressed intestinal permeability and improved IBD symptoms by enhancing wound healing in an IBD rodent model^7^. Protein metabolism in intestinal mucosa is essential for gut homeostasis and maintenance of the epithelial barrier^13^. L-Arg increases intestinal protein synthesis and epithelial repair by activating the mechanistic target of rapamycin (mTOR) signaling pathway^14^. Once activated, mTOR phosphorylates its downstream targets, ribosomal protein S6 kinase (p70 S6K) and eukaryotic initiation factor 4E-binding protein 1 (4E-BP1), thereby promoting mRNA translation, protein synthesis, and cell growth^15^. In contrast, blockage of the mTOR pathway suppresses intestinal cell migration^16^. In this way, L-Arg contributes to wound healing and protein synthesis, while significantly enhancing mTOR signaling; this pathway may be a promising agent in intestinal wound closure.

**Figure 1:**
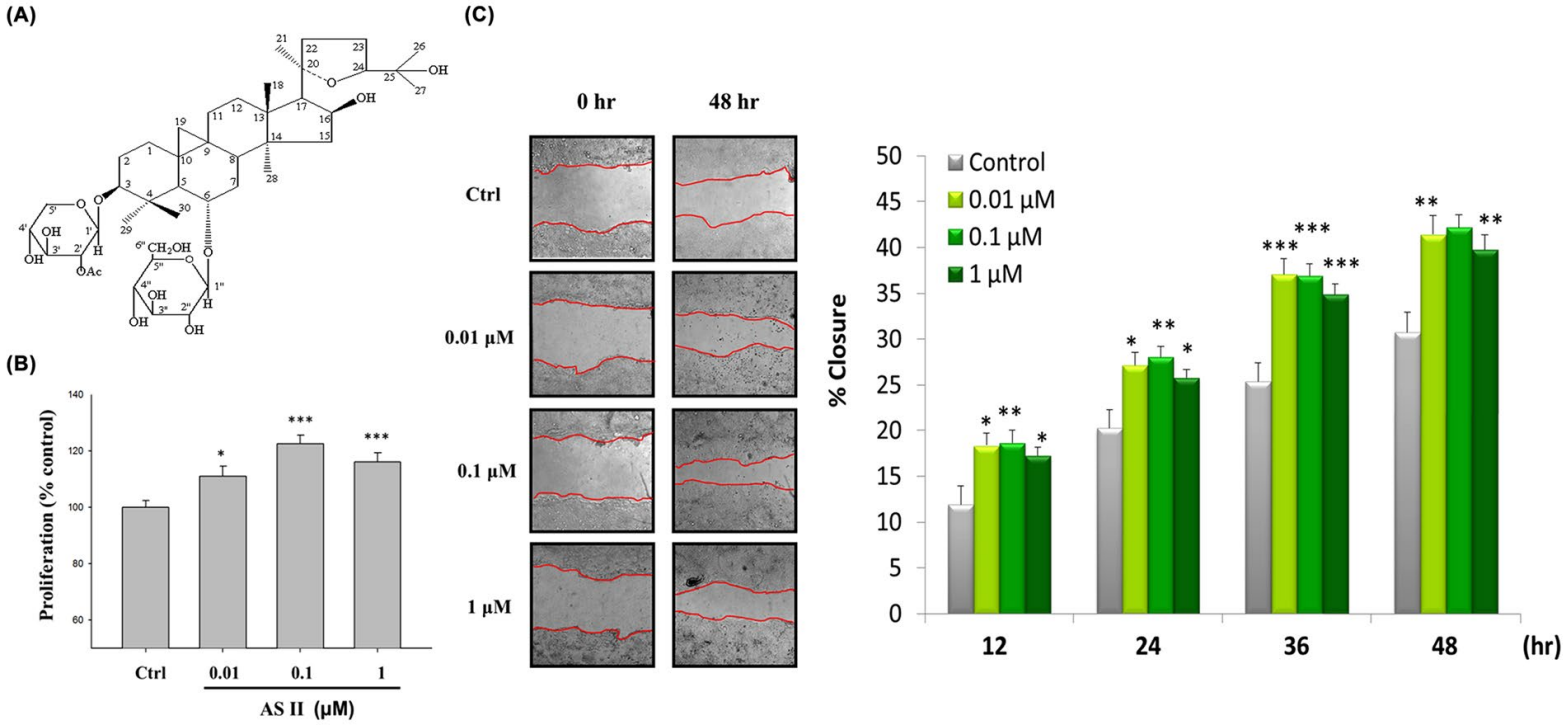
Effects of astragaloside II (AS II) on cell proliferation and scratch wound closure in Caco-2 cells. (**A**) Chemical structure of AS II. (**B**) Caco-2 cells were treated with 0.01, 0.1, and 1 μM AS II for 24 hr. Cell proliferation was measured using a CCK-8 kit. (**C**) The Caco-2 cell monolayers were scratched and incubated with the indicated concentrations of AS II. Wound closure was photographed and quantified over time. Data represent mean ± SEM (n = 6). *p < 0.05, **p < 0.01, and ***p < 0.001 versus the untreated control.

Radix Astragali is a well-known medicinal herb for reinforcing Qi (the vital energy) in traditional Chinese medicine, which considers it to possess immunomodulatory, wound-healing, anti-inflammatory, anti-aging, anti*-*oxidant, and hypoglycemic properties^17^. *Astragalus membranaceus* contains a variety of compounds, including polysaccharides, flavonoids, and saponins. Astragaloside II (AS II; Fig. 1A) is one of the major cycloartane-type triterpene glycosides extracted from Radix Astragali^18^, and has recently been reported to be a potential adjunctive agent in cancer chemotherapy^19^, enhancement of osteogenesis^20^, and modulation of T cell activation^21^. However, the effects and underlying mechanism of AS II on intestinal wound healing are unknown. In the present study, we examined the effect of AS II in repair and restoration of intestinal epithelial barrier function and the signaling mechanism involved, both *in vitro* and *in vivo*.

## Results

### AS II promotes cell proliferation and scratch wound closure in Caco-2 cells

We first investigated the effect of AS II on cell proliferation. AS II increased cell proliferation (fold-change compared with control: 1.11 ± 0.03 for 0.01 μM, *p* < 0.05; 1.22 ± 0.03 for 0.1 μM, *p* < 0.001; 1.16 ± 0.03 for 1 μM, *p* < 0.001) (Fig. 1B). AS II improved scratch wound closure in Caco-2 cells in a time-dependent manner (Fig. 1C); the maximum effect of scratch wound closure was observed at 0.1 μM. Forty-eight hours after initiating the scratch wound assay, scratch wound percent closure increased from 30.77 ± 2.13 to 41.42 ± 2.09 (0.01 μM, *p* < 0.01), 42.17 ± 1.42 (0.1 μM, *p* < 0.001), and 39.79 ± 1.61 (1 μM, *p* < 0.01) (Fig. 1C).

### AS II increases L-Arg uptake and CAT protein levels in Caco-2 cells

**Figure 2:**
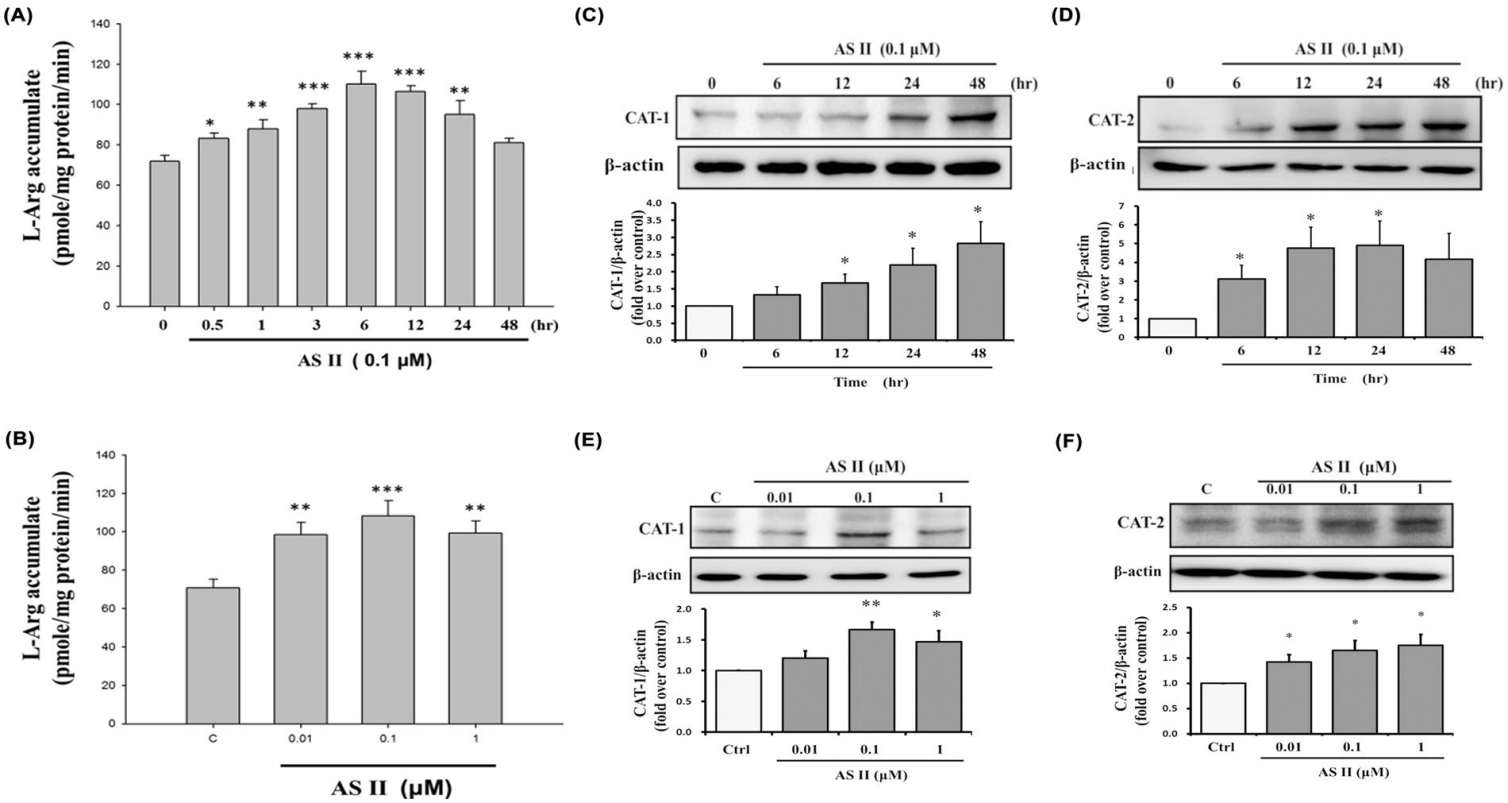
Effects of astragaloside II (AS II) on L-arginine (L-Arg) uptake and cationic amino acid transporter (CAT) expression. Differentiated Caco-2 cells were incubated with AS II and then analyzed for L-Arg accumulation at the indicated time periods (**A**) and concentrations (**B**). CAT1 and CAT2 protein levels in Caco-2 cells were assessed by western blotting at different time points (**C** and **D**) and concentrations (**E** and **F**) of AS II treatment. Data represent mean ± SEM (n = 6 for L-Arg uptake, n = 3 for western blotting). *p < 0.05, **p < 0.01, and ***p < 0.001 versus the untreated control.

To assess the effects of AS II on L-Arg uptake, we treated Caco-2 cells with AS II. As shown in Fig. 2A, 0.1 μM of AS II significantly increased L-Arg cellular uptake between 0.5 and 24 hr compared with the control, reaching 104.73 ± 3.90 pmol/mg protein/min 6 hr after treatment (*p* < 0.001). The stimulatory effect of AS II was observed in the concentrations tested; the greatest effect was seen in the 0.1-μM treatment (108.18 ± 8.02 pmol/mg protein/min, *p* < 0.001 compared with the control; Fig. 2B). To identify the L-Arg transporters involved, we measured the expression of CAT1 and CAT2, and found that 0.1 μM AS II significantly increased both CAT1 and CAT2 expression (Fig. 2C and D) between 6 and 48 hr after treatment. A higher concentration of AS II (1 μM) also increased CAT1 and CAT2 protein levels (1.45 ± 0.18 fold-increase over control for CAT1, *p* < 0.05; 1.55 ± 0.05 fold-increase over control for CAT2, *p* < 0.05; Fig. 2E and F).

### AS II activates the mTOR pathway and enhances protein synthesis in Caco-2 cells

**Figure 3:**
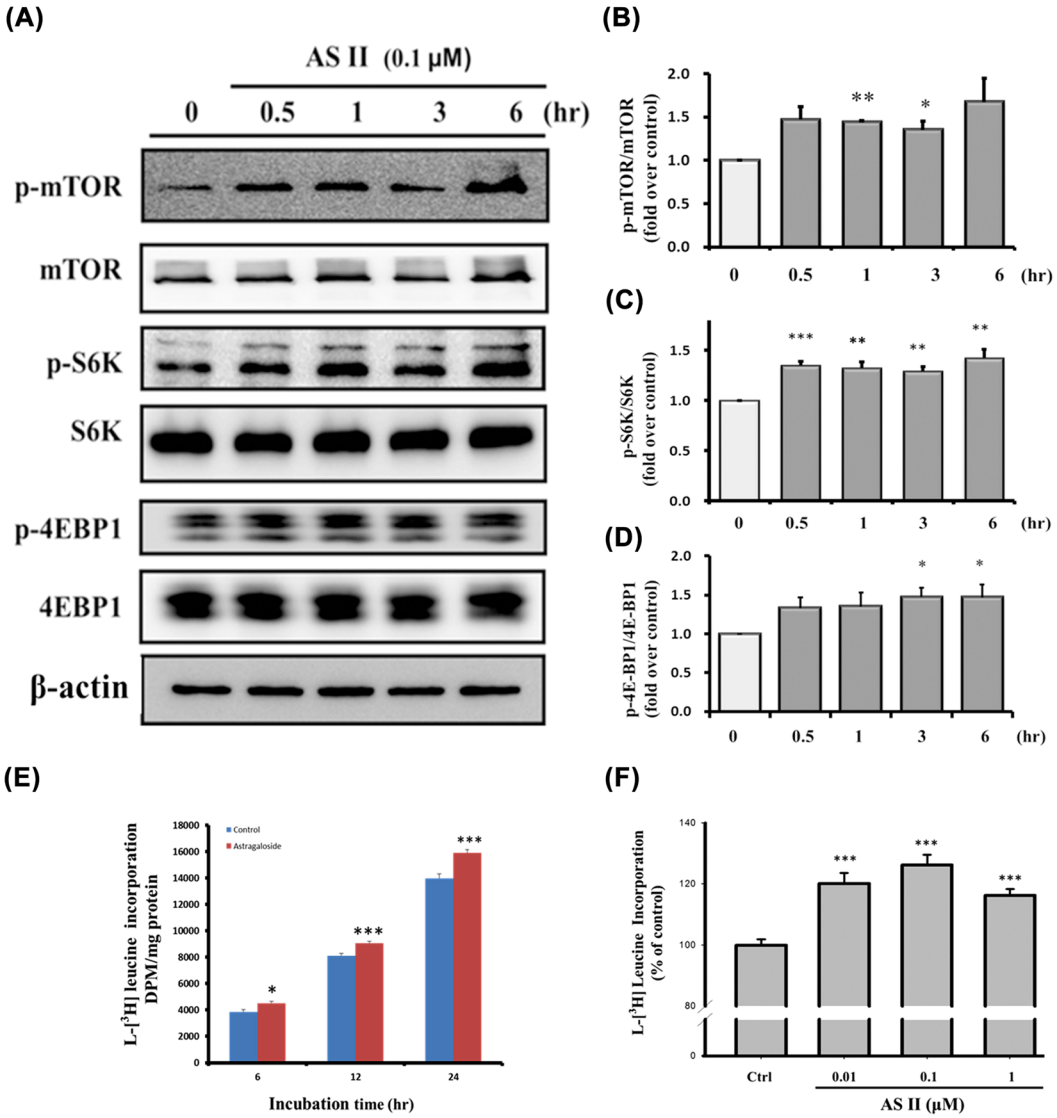
Effects of astragaloside II (AS II) on the mTOR signaling pathway and on protein synthesis. Caco-2 cells were incubated with 0.1 μM AS II and then harvested for western blotting (**A**). Levels of p-mTOR/mTOR (**B**), p-S6K/S6K (**C**), and p-4E-BP1/4E-BP1 (**D**) were quantified. Protein synthesis was assayed by [^3^H]-leucine incorporation and quantified by time (**E**) and concentration (at 24 hr, **F**). Data represent mean ± SEM (n = 3). *p < 0.05, **p < 0.01, and ***p < 0.001 versus the untreated control.

We studied the effect of AS II on mTOR activation and protein synthesis. AS II (0.1 μM) increased phosphorylated mTOR levels, especially 1 hr after treatment (1.44 ± 0.02 fold-increase over control, *p* < 0.01; Fig. 3A and B). AS II also promoted the phosphorylation of mTOR’s downstream targets S6K and 4EBP1, especially 3 and 6 hr after treatment (*p* < 0.01 for p-S6K and *p* < 0.05 for p-4EBP1 compared with the control; Fig. 3A,C and D). We then investigated the effect of AS II on cellular protein synthesis. AS II significantly increased protein synthesis in both a time- and concentration-dependent manner (Fig. 3E and F). These findings indicate AS II increases both the mTOR signal pathway and protein synthesis in Caco-2 cells.

### Lysine and rapamycin both suppress the effects of AS II on the mTOR signaling pathway

**Figure 4:**
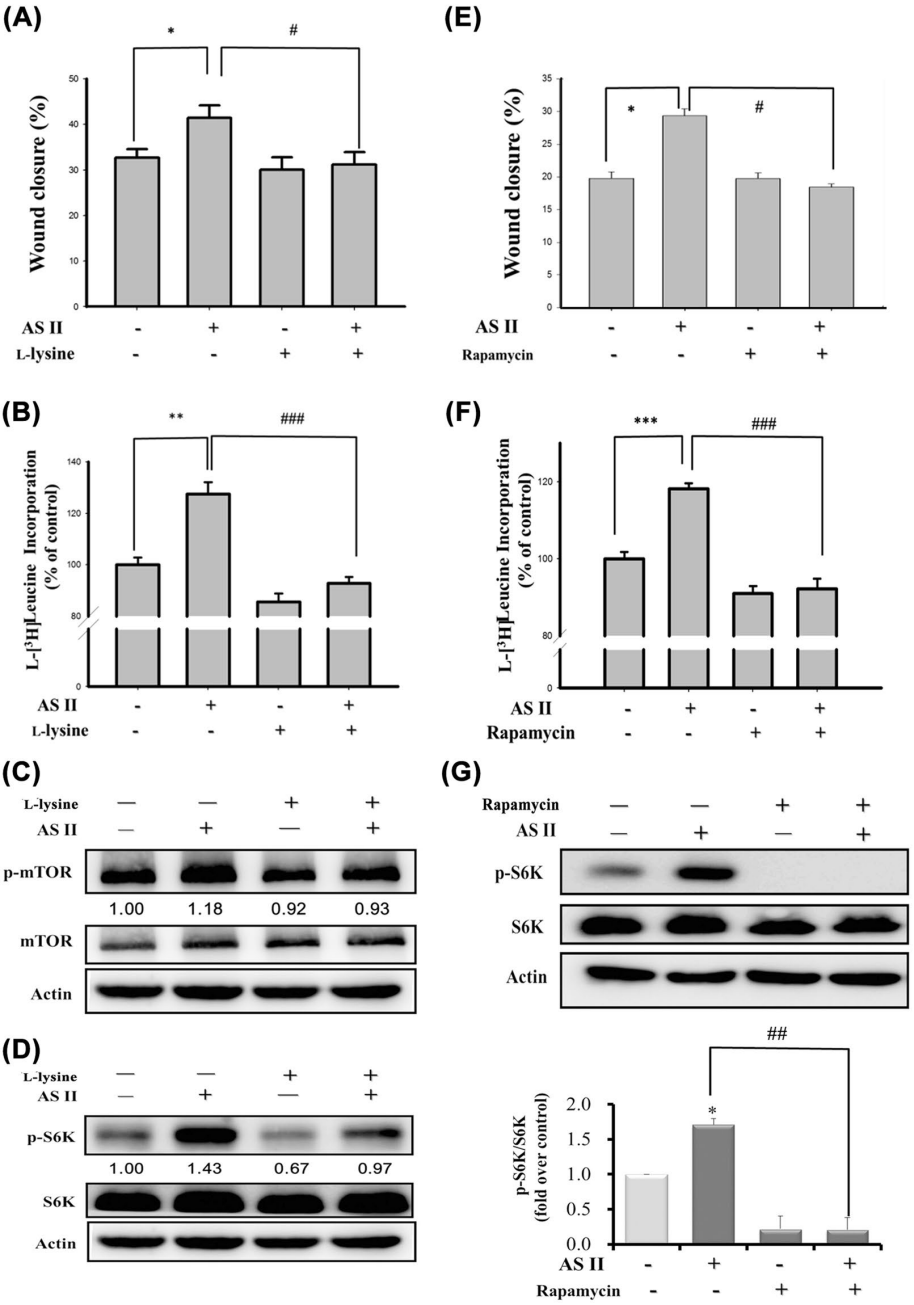
Effects of L-lysine and rapamycin on wound closure and protein synthesis in astragaloside II (AS II)-treated Caco-2 cells. Cells were incubated with L-lysine (20 mM) or rapamycin (100 nM) 30 min prior to treatment with 0.1 μM AS II. Scratch wound closure and protein synthesis were quantified after 36 hr (**A**) and 24 hr (**B**), respectively. Levels of p-mTOR/mTOR (**C**) and p-S6K/S6K (**D**) were analyzed in the L-lysine-treated cells. Similarly, scratch wound closure (**E**), protein synthesis (**F**), and p-S6K/S6K levels (**G**) were analyzed in the rapamycin-treated cells. Data represent mean ± SEM (n = 3). *p < 0.05 and **p < 0.01 versus the untreated control; ^#^ *p* < 0.05 versus the AS II-treated cells.

To investigate the roles of L-Arg uptake and mTOR signaling in the wound-healing activity of AS II, we used lysine (a competitive inhibitor of CAT1 and CAT2 for L-Arg uptake) and rapamycin (the mTORC1 inhibitor). Pretreatment with lysine reduced AS II-upregulated scratch wound closure (from 40.58 ± 1.48 to 32.10 ± 1.54%; *p* < 0.01; Fig. 4A), protein synthesis (from 120.60 ± 2.48 to 94.88 ± 2.09%; *p* < 0.001; Fig. 4B), p-mTOR (from 1.18 to 0.93 fold-increase over control; Fig. 4C), and p-S6K (from 1.43 to 0.97 fold-increase over control; Fig. 4D). Rapamycin exerted similar effects on AS II-upregulated scratch wound closure (from 29.36 ± 1.01 to 18.43 ± 0.52%; *p* < 0.05; Fig. 4E), protein synthesis (from 118.14 ± 1.49 to 92.20 ± 2.56% compared with control; *p* < 0.001; Fig. 4F), and p-S6K (from 1.71 ± 0.08 to 0.21 ± 0.17 fold-increase over control; *p* < 0.01; Fig. 4G). The results indicated that both upregulation of the L-Arg uptake and mTOR activation are necessary for AS II-mediated wound healing.

### AS II attenuates 2,4,6-trinitrobenzene sulfonic acid (TNBS)-induced colitis in mice

**Figure 5:**
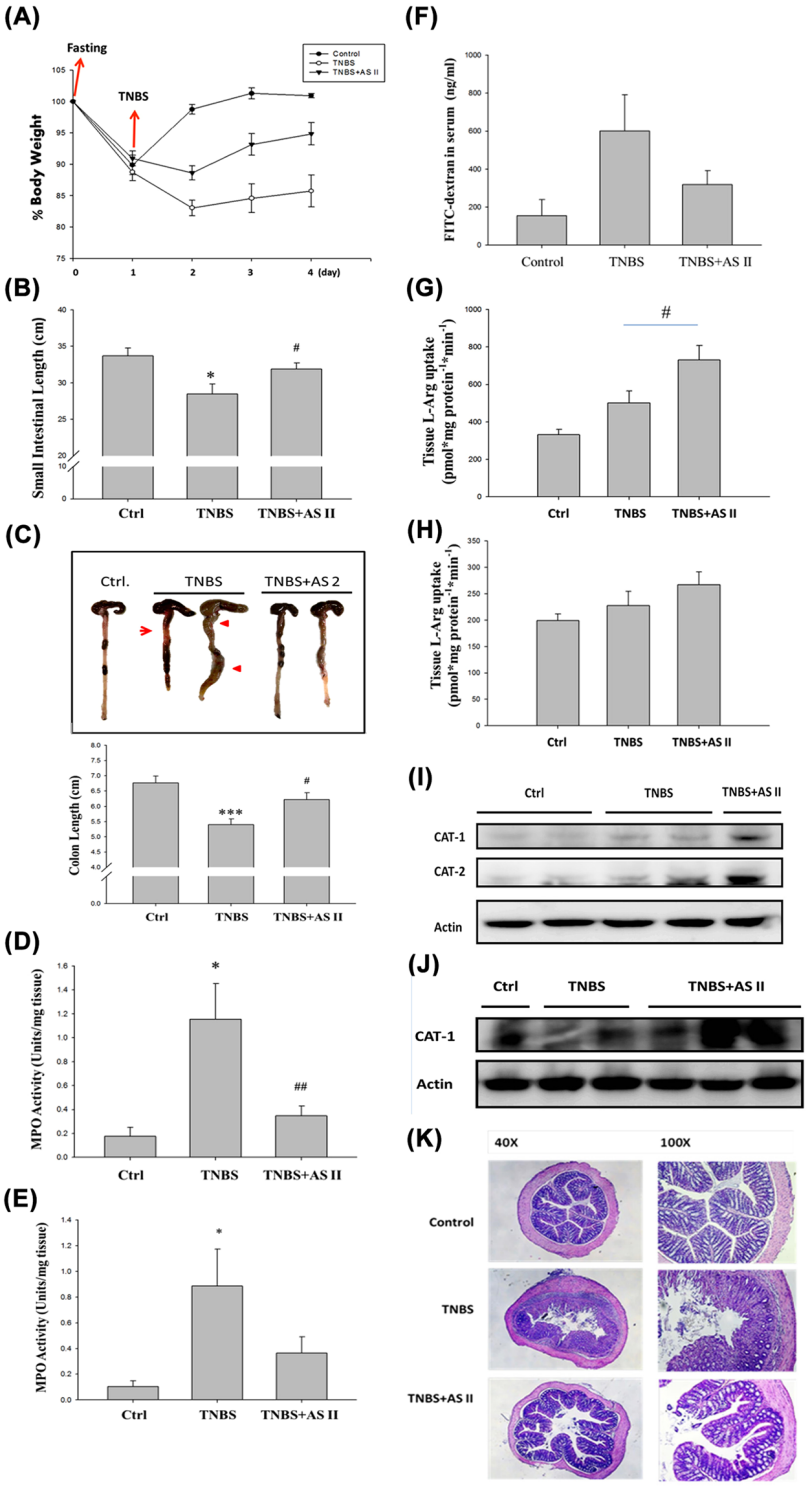
Effects of astragaloside II (AS II) on a 2,4,6-trinitrobenzene sulfonic acid (TNBS)-induced mouse model of colitis. (**A**) Body weight. (**B**) Length of the small intestine. (**C**) Length of the colon. Myeloperoxidase (MPO) activity was examined in both the small intestine (**D**) and the colon (**E**). (**F**) mucosal permeability. (**G** and **H**) L-arginine (L-Arg) uptake. (**I** and **J**) Levels of cationic amino acid transporters (CATs). (**K**) Histological examination was performed by photomicrography (original magnification at 40× and 100×). Data represent mean ± SEM (n = 6). *p < 0.05, ***p < 0.01, and ***p < 0.001 versus the untreated control; ^#^ *p* < 0.05 and ^##^ *p* < 0.01 versus the AS II-treated cells.

To assess the *in vivo* effects of AS II, we established a TNBS-induced mouse model of colitis. We found that treatment with AS II attenuated TNBS-induced weight loss (from 85.75 ± 2.54 to 94.85 ± 1.78% compared with the control [100%], *p* < 0.01; Fig. 5A), and affected the length of the small intestine (from 28.46 ± 1.40 to 31.88 ± 0.85 cm, *p* < 0.05; Fig. 5B), length of the large intestine (from 5.40 ± 0.18 to 6.22 ± 0.22 cm, *p* < 0.05; Fig. 5C), and myeloperoxidase (MPO) activity in the small intestine (from 1.15 ± 0.30 to 0.35 ± 0.08 unit/mg protein, *p* < 0.01; Fig. 5D). MPO activity in the large intestine (Fig. 5E) and epithelial barrier permeability (Fig. 5F) were also measured, but did not differ statistically from the control. AS II significantly increased L-Arg uptake in the small intestine (from 500.60 ± 64.17 to 731.29 ± 75.82 pmol/min/mg protein, *p* < 0.05; Fig. 5G). Slight but non-significant increase was observed in the colon (Fig. 5H). AS II also increased CAT1 and CAT2 levels in the small intestine (Fig. 5I) and CAT1 levels in the colon (Fig. 5J). Colon histology appeared altered (Fig. 5K).

## Discussion

**Figure 6:**
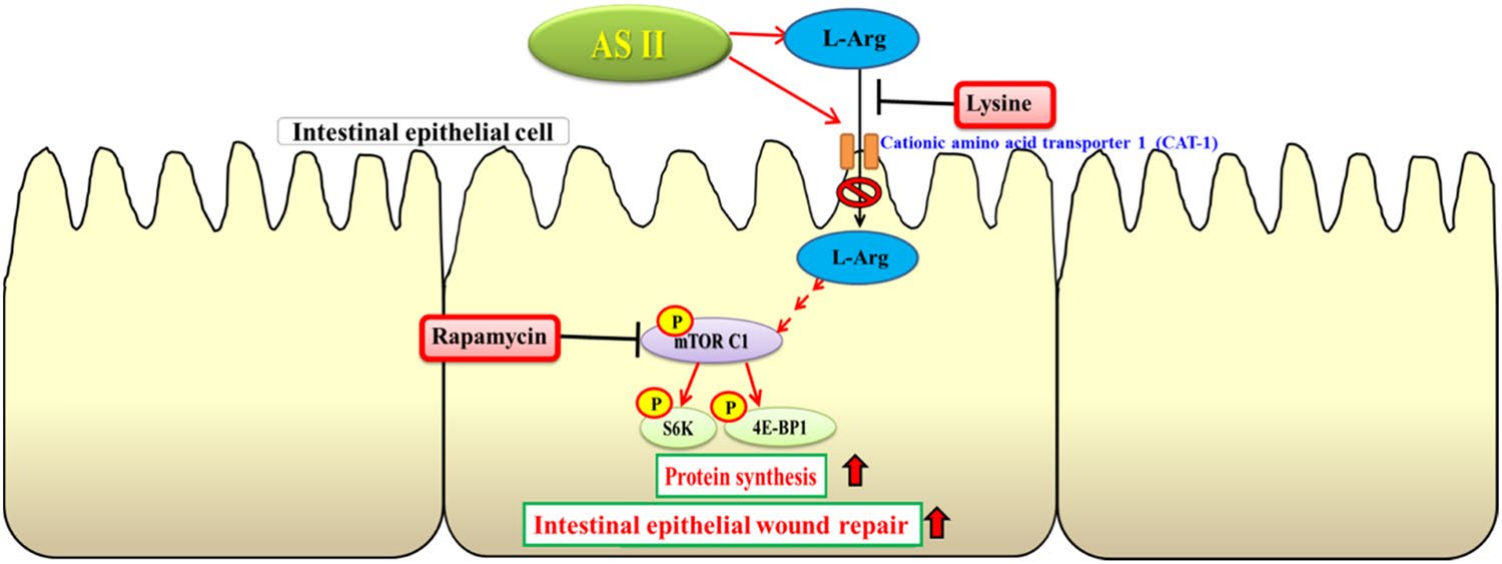
Proposed mechanism of astragaloside II (AS II) action on intestinal epithelial wound repair.

In this study, we examined the effects of AS II, one of the major bioactive components of *A*. *membranaceus*, on repair and restoration of intestinal epithelial barrier function, and investigated the signaling mechanism involved. We found that AS II can promote scratch wound closure, cell proliferation, and arginine uptake, and can induce the arginine transporters CAT1 and CAT2 in differentiated human intestinal Caco-2 cells. In addition, AS II enhanced the phosphorylation of mTOR, S6K, and 4E-BP1, while protein synthesis increased significantly. The effects of AS II on wound closure were also confirmed using L-lysine (a competitive inhibitor of L-Arg uptake) and rapamycin (a specific mTOR inhibitor), as both of these inhibitors suppressed the effect of AS II on wound closure. These results suggest that L-Arg uptake and mTOR signaling activation are involved in AS II-induced wound healing. The effect of 0.1 μM AS II was greater than that of 1 μM AS II on cell proliferation, wound closure, L-Arg uptake, CAT1 expression, and leucine incorporation. These findings indicate that 0.1 μM is an effective concentration. A 1-μM treatment appeared to exert adverse effects or to inhibit the beneficial effects. The higher concentration may induce or interfere with certain cellular activities. The dose independent activity has been reported in different natural product^22^. In a TNBS-induced mouse colitis model, AS II was shown to ameliorate the severity of colitis symptoms such as weight loss, reduction in intestinal length, intestinal inflammation, and increased mucosal permeability. AS II also increased intestinal L-Arg uptake and mucosal CAT protein levels. Our findings indicate that AS II promotes intestinal epithelial healing through increased L-Arg uptake and protein synthesis, which are likely mediated by the increased expression of L-Arg transporters and activation of the mTOR signaling pathway, respectively. Figure 6 displays our proposed mechanism of AS II action.

A recent strategy for managing IBD has been to improve the integrity of the intestinal epithelial barrier and prevent recurrence of intestinal inflammation, especially in Crohn’s disease^23^. In traditional Chinese medicine, Radix Astragali is a tonic herb used in a variety of inflammatory and immune diseases, as well as for wound healing^21^. In the present study, we showed that AS II can promote intestinal epithelial wound healing via the mTOR pathway *in vitro* and *in vivo*. IBD pathogenesis is a complex process, which involves the immune response and inflammation^1^. Crude extract of Radix Astragali has been shown to exert protective and anti-inflammatory effects in several animal models of colitis^24–26^. To our knowledge, this is the first study to report the beneficial effects of AS II, a major bioactive component of *Astragalus*, in a TNBS-induced animal model of colitis. Our findings provide a better understanding of the therapeutic potential of Radix Astragali in IBD. We also showed that MPO activity was inhibited in AS II-treated mice, indicating its anti-inflammatory potential. Future work should assess whether AS II can attenuate IBD symptoms by modulating the immune response.

*Astragalus* products have been used as immunomodulatory and wound-healing agents in traditional Chinese medicine^17^. However, the detailed mechanisms and bioactive components are largely unknown. Radix Astragali contains a variety of compounds such as polysaccharides, flavonoids, isoflavonoids, and polyphenols. Among these, astragalosides have been shown to promote wound-healing activity *in vitro* and *in vivo* ^27^. To date, most studies have focused on the pharmacological effects of AS IV, a major astragaloside, which has been shown to improve wound repair and diminish scarring in burn wounds^28^. However, few studies have investigated AS II’s effects on intestinal epithelial healing. The structure of AS II is similar to that of AS IV, apart from an additional acetyl group at C-2 of the xylose residues. We found that AS II improved intestinal epithelial repair by enhancing L-Arg uptake and activating the mTOR pathway. AS II has been shown to exert better T cell immune-stimulating effects than AS IV^21^. This suggests that AS II may be a more potent immune stimulator than AS IV. Current drugs indicated for IBD treatment include anti-inflammatory drugs, immunosuppressives, antibiotics, and biologic agents. These agents can cause side effects such as gastrointestinal symptoms, hepatotoxicity, and renal toxicity, limiting their clinical applications^29^. Radix Astragali is considered a top-grade herb in traditional Chinese medicine, meaning a safe and nontoxic herbal medicine^30^. Astragalus supplementation (90 g daily) has been reported to have low toxicity and no significant adverse effects^31^. AS II may therefore be a safe and promising compound for treating IBD or immune disorders.

L-Arg has been shown to promote wound healing in skin, intestinal epithelial tissues, and *in vivo* IBD models^7,32,33^. In the present study, AS II improved L-Arg uptake in both cultured Caco-2 cells and colonic tissue, and increased CAT1 and CAT2 levels. Cellular uptake of L-Arg through CAT2 is important for colonic epithelial wound repair^10^. One study indicated that 12-O-tetradecanoylphorbol-13-acetate increased L-Arg uptake and CAT levels by activating the protein kinase C (PKC) signaling pathway in intestinal epithelial cells^34^. Whether AS II promotes CAT expression via the PKC signaling pathway requires further study. High doses of L-Arg supplementation (500 mg/day) exacerbated colonic inflammation and fibrosis in rats because of excessive nitric oxide production and collagen deposition^35^. A study in humans^36^ reported that serum levels of L-Arg were 150% higher in patients with ulcerative colitis than in healthy individuals; levels of ornithine and lysine, which are also transported by CATs, were elevated as well, leading to competitive inhibition of arginine uptake. In the present study, AS II increased cellular L-Arg uptake without L-Arg supplementation. These findings suggest that enhancement of cellular L-Arg uptake, rather than L-Arg supplementation, may be a better strategy for treating IBD. L-glutamine supplementation has been shown to contribute to beneficial effects in a dextran sulfate sodium-induced colitis model^37^. The effect of AS II on L-glutamine absorption warrants further study.

L-Arg-mediated activation of the mTOR pathway improves intestinal cell migration and epithelial wound healing^14^, and was confirmed in genetically defined mouse models^38^. We found that AS II activated mTOR and its downstream targets, S6K and 4E-BP1. AS II increased protein synthesis in a concentration- and time-dependent manner, contributing to accelerated cell proliferation and wound healing. We confirmed the findings using lysine, a competitive inhibitor of L-arg uptake and rapamycin, a specific mTOR inhibitor. The mTOR signaling pathway is activated by several amino acids, including L-Arg and L-leucine^39,40^. Future studies should also assess the effect of AS II on other amino acids, such as L-leucine. In addition, the CATs protein levels may not be equal to the activity transport capacity. Our results show L-Arg uptake peaks at 6 h that are consistent with the previous study that the protein kinase C (PKC) activator 12-O-tetradecanoylphorbol 13-acetate (TPA), stimulated system y + arginine transport activity in Caco-2 cells with transport capacity (Vmax) between 6–12 h^34^. In addition, absorption of cationic amino acids is largely dependent on y^+^ transport system, such as CAT1 and CAT2^41^. It suggests AS II might promote the activity of CATs via the PKC pathway. However, the relationship between CATs levels and activity requires further investigation.

In conclusion, AS II promotes intestinal epithelial repair by enhancing L-Arg uptake and activating the mTOR pathway. These findings suggest that AS II may be effective in relieving colitis.

## Methods

### Materials

*A*. *membranaceus* var. *mongholicus* was authenticated by Dr. H.C. Lin at the National Defense Medical Center, where a voucher specimen (NDMCP no. 1000901) has been deposited. L-Arg, L-lysine (Sigma-Aldrich, St. Louis, MO, USA), L-[^3^H]-Arg, L-[^3^H]-leucine (American Radiolabeled Chemicals, St. Louis, MO, USA), and Ultima Gold liquid scintillation cocktails (PerkinElmer, Waltham, MA, USA) were used in the study.

### Preparation of AS II

Dried root powder of Radix Astragali (9.5 kg) was soaked in 95% ethanol (20 L × 7), yielding a 756.25-g solution of Radix Astragali extract with evaporation under reduced pressure. The extract was then partitioned between n-BuOH-H_2_O and n-hexane-90% MeOH to yield 90% MeOH layer (334.68 g). The 90% MeOH fraction was subjected to medium-pressure liquid chromatography and eluted with an H_2_O-MeOH gradient system, yielding three fractions. Fraction 2 (83.93 g) was further chromatographed on silica gel and eluted with CHCl_3_-MeOH-H_2_O (10:5:1) to yield AS II (1.68 g), which was identified using spectral data in the literature (Fig. 1A)^42,43^.

### Cell culture

The human intestinal epithelial cell line Caco-2, a widely used model for studying the intestinal barrier, permeability, and wound healing^44^, was purchased from the American Type Culture Collection (ATCC, Manassas, VA, USA) and maintained in Dulbecco’s modified Eagle’s medium (DMEM) with regular supplements.

### Scratch wound assay

The assay was performed as described previously^45^. Briefly, Caco-2 cells were seeded in 24-well plates (2 × 10^5^ cells/well) and allowed to reach confluence. Scratch wounds were made using a sterile 10-μL pipette tip to create a straight cell-free line simulating a wound. After scratching, the cells were rinsed gently with phosphate-buffered saline (PBS) to remove detached cells. Next, cells were incubated with AS II in 5% CO_2_ at 37 °C. Cell migration was measured using photomicrography equipment (Leica Microsystems, Wetzlar, Germany) to compare the wound area 0, 12, 24, 36, and 48 hr after making the scratch.

### Cell proliferation assay

Cell viability was assessed using a cell counting kit (CCK-8, Dojindo, Kumamoto, Japan) as described previously^46^. Briefly, Caco-2 cells (5 × 10^3^ cells/well) were seeded in 96-well plates and incubated in serum-free medium with various concentrations of AS II for 48 hr. The medium was then removed, and 10 μL of CCK-8 in 90 μL of PBS were added to the cells for 2 hr. Absorbance was measured at 450 nm using a microplate reader (Molecular Devices, Sunnyvale, CA, USA).

### L-Arg uptake assay

The assay was carried out as described previously^11^. Briefly, Caco-2 cells (2.5 × 10^4^ cells/well) were seeded in 24-well plates. Cells were cultured for 7 days after reaching confluence to allow differentiation. The cells were then rinsed three times with transport buffer and incubated in transport buffer containing 0.1 mM L-Arg and 1 μCi/mL L-[^3^H]-Arg for 5 min. The buffer was then removed; cells were rinsed three times with cold PBS and dissolved in 300 μL lysis buffer (1 N NaOH) for 1 hr at room temperature. Tissue L-Arg uptake measurement was performed as described previously^7^. A 1.5-cm section of intestinal or colonic tissue was dissected and immediately incubated in transport buffer containing 0.1 mM L-Arg and 1 μCi/mL L-[^3^H]-Arg for 5 min. The transport buffer was then removed; the tissues were rinsed three times with cold PBS and then lysed in 500 μL 0.1 NHNO_3_ by gently shaking for 24 hr at room temperature. A 200-μL aliquot of the lysate was then collected and mixed with 2 mL of liquid scintillation cocktails. Radioactivity was measured with a scintillation counter (TopCount, Packard BioScience, Meriden, CT, USA). L-Arg uptake is expressed as nmol/mg of protein/min.

### *De novo* protein synthesis assay

The assay was described in a previous study^47^. Briefly, Caco-2 cells were seeded in 24-well plates (5 × 10^4^ cells/well) and cultured for 7 days after reaching confluence. Cells were incubated in DMEM containing 1 μCi/ml [^3^H]-leucine for 4 hr and then rinsed three times with cold PBS. Next, cells were dissolved in 200 μL lysis buffer and precipitated with 10% trichloroacetic acid (TCA) for 10 min. After centrifugation at 12,000 *g* for 5 min, the supernatant was discarded and the protein pellet was rinsed with 10% TCA and then dissolved in 300 μL NaOH (1 N). The solution (200 μL) was mixed with 2 mL of liquid scintillation cocktails. Protein synthesis was measured with a scintillation counter (TopCount, Packard BioScience).

### Animal study protocol

Male C57BL/6JNarl 5-week-old (18–22 g) mice were purchased from the National Laboratory Animal Center (Taipei, Taiwan). Animal feeding and experimental procedures were approved by the Institutional Animal Care and Use Committee of the National Defense Medical Center (certificate number: IACUC-13–302) and performed in accordance with the relevant guidelines and regulations^48^. The mice were gavaged with 1 mL saline or AS II (10 mg/kg) once daily for 11 consecutive days. TNBS (100 mg/kg) was administered via the rectum on the 8th day^49^. The mice were anesthetized with pentobarbital sodium (i.p. 70 mg/kg), and blood samples were collected from the orbital sinus. At the end of the experiment (11th day), the animals were euthanized using CO_2_ to allow harvesting of tissues. The intestine and colon were dissected.

### MPO activity in colon tissue

MPO activity was assayed as previously described^50^. In brief, the tissue was lysed and freeze-thawed for three cycles in extraction buffer (1:20, w/v). Homogenates were then centrifuged at 14,000 rpm for 20 min. Ten microliters of supernatant were collected and mixed with 190 μL assay buffer (1.68 mM 3,3′,5,5′-tetramethylbenzidine and 0.00015% hydrogen peroxide). MPO activity was determined at 650 nm using a microplate reader (Molecular Devices).

### Intestinal permeability assay

The assay was performed as described previously^51^. Briefly, the mice were fasted for 8 hr and then gavaged with FITC-dextran (50 mg/100 g; Sigma-Aldrich) 4 hr after fasting. Blood samples were collected from the retro-orbital sinus and centrifuged at 12,000 *g* for 20 min. Serum was measured at excitation and emission wavelengths of 490 and 520 nm, respectively, using a fluorescence microtiter plate reader (POLARstar Galaxy; BMG Labtech, Ortenberg, Germany).

### Western blot analysis

This method was described in our previous study^48^. Cells were plated in 6-well plates (1 × 10^6^ cells/well) and treated accordingly. Cells were then harvested in 0.2 mL of RIPA lysis buffer, separated by 10% SDS-PAGE, and transferred to PVDF membranes (Millipore, Billerica, MA, USA). Immunoblotting was performed using primary antibodies against CAT1, CAT2 (Abcam, Cambridge, UK), p-mTOR, mTOR, p-p70S6K, p70S6K, p-4E-BP1, 4E-BP1 (Cell Signaling Technology, Beverly, MA, USA), and the housekeeper β-actin (Millipore). The signals were visualized with an enhanced chemoluminescence kit (Amersham Biosciences, Little Chalfont, UK) followed by exposure of the blots to X-ray film.

### Histology

A histological examination was performed following a method described previously^50^. Intestinal tissues were soaked in 10% formaldehyde solution for 24 hr, then stained with hematoxylin and eosin.

### Statistical analysis

All data represent the mean ± SEM. Significant differences between group means were determined by one-way ANOVA followed by a Bonferroni post hoc test using SPSS version 22 (IBM SPSS, Armonk, NY, USA). *p* < 0.05 was considered statistically significant.

## Electronic supplementary material

Supplementary information
