# Supplementary material for: Astragaloside II promotes intestinal epithelial repair by enhancing L-arginine uptake and activating the mTOR pathway
Source: Sci Rep. 2017 Sep 26;7:12302. doi: 10.1038/s41598-017-12435-y (PMC5614914; doi:10.1038/s41598-017-12435-y)

**Astragaloside II promotes intestinal epithelial repair by enhancing L-arginine uptake and activating the mTOR pathway**

Shih-Yu Lee^1^, Wei-Cheng Tsai^2^, Jung-Chun Lin^3^, Blerina Ahmetaj-Shala^4^, Su-Feng Huang^2^, Wen-Liang Chang^5^, Tsu-Chung Chang^2,*^

^1^Graduate Institute of Aerospace and Undersea Medicine, National Defense Medical Center, Taipei, Taiwan.

^2^Department of Biochemistry, National Defense Medical Center, Taipei, Taiwan

^3^Division of Gastroenterology and Hepatology, Department of Internal Medicine, Tri-Service General Hospital, National Defense Medical Center, Taipei, Taiwan;

^4^National Heart & Lung Institute, Imperial College London, London, United Kingdom.

^5^School of Pharmacy, National Defense Medical Center, Taipei, Taiwan

Authors e-mail Address:

Shih-Yu Lee: [leeshihyuno1@mail.ndmctsgh.edu.tw](mailto:leeshihyuno1@mail.ndmctsgh.edu.tw);

Wei-Cheng Tsai: [j4418m@gmail.com](mailto:j4418m@gmail.com);

Jung-Chun Lin: doc10506@gmail.com

Blerina Ahmetaj-Shala: b.ahmetaj@imperial.ac.uk;

Su-Feng Huang: kate@nuliv.com

Wen-Liang Chang: wlchang@mail.ndmctsgh.edu.tw

Tsu-Chung Chang: [tcchang@mail.ndmctsgh.edu.tw](mailto:tcchang@mail.ndmctsgh.edu.tw)

**RUNNING TITLE:** Astragaloside II promotes intestinal epithelial repair

Corresponding author: Tsu-Chung Chang: Department of Biochemistry, National Defense Medical Center, *P.O. Box 90048-501*, Neihu 114, *Taipei, Taiwan, R.O.C.* Tel: 886-2-87923100 ext. 18820. Fax: 886-2-87924820. E-mail: tcchang@mail.ndmctsgh.edu.tw

Figure 1

(A)

(B)


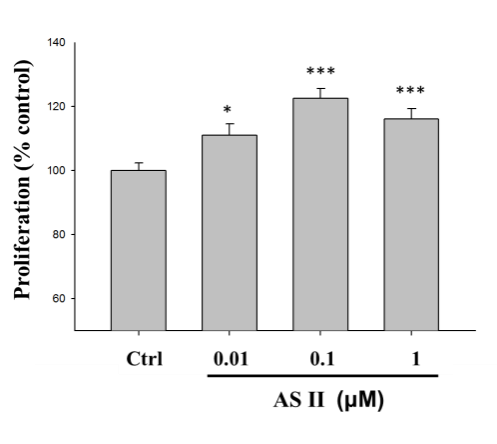


(C)


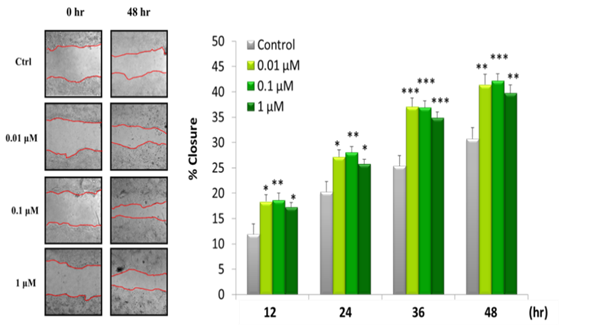


Figure 2

(A)


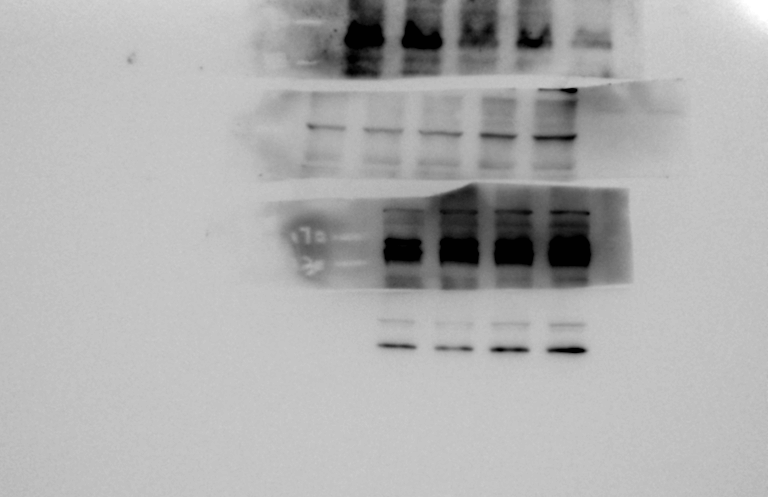

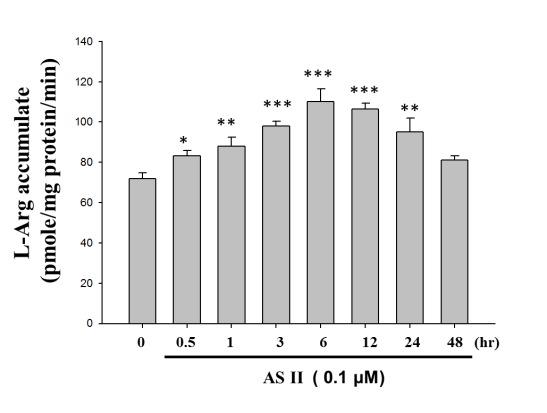


(B)


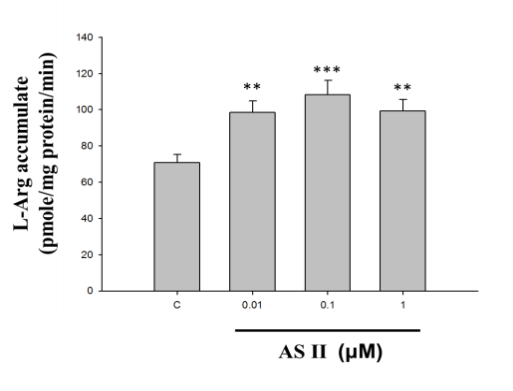


CAT-1


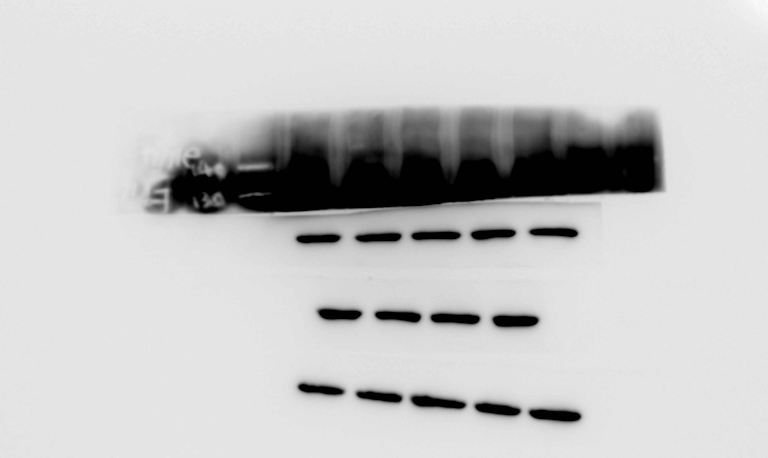
(C)


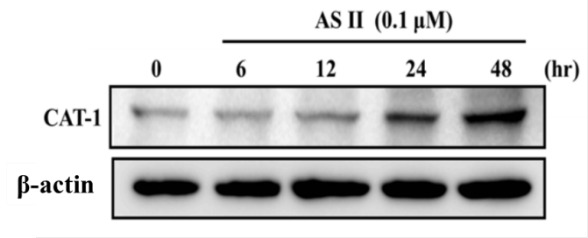


β-actin


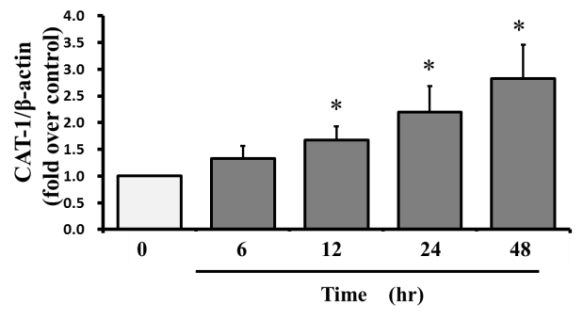


(D)


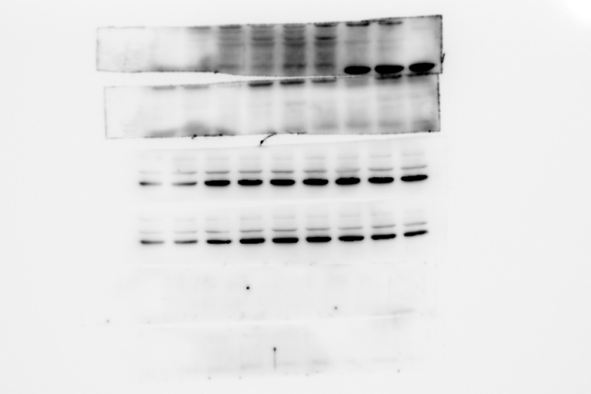

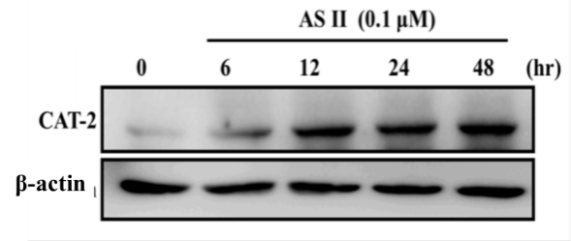


CAT-2


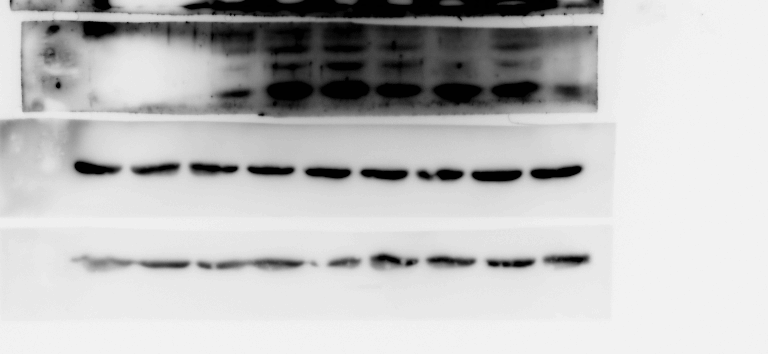

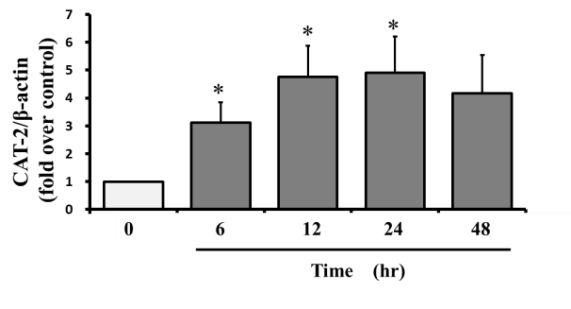


β-actin

(E)


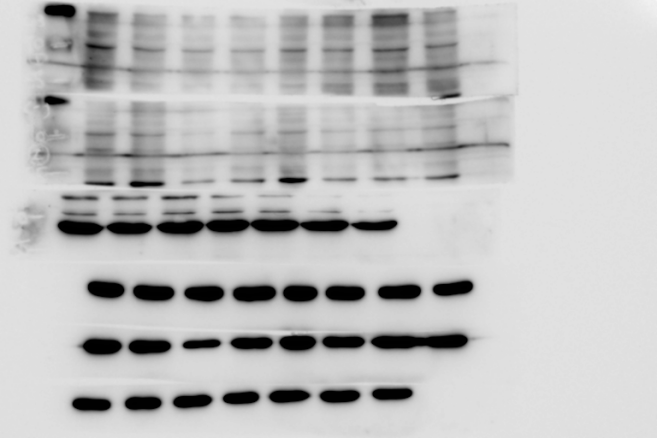

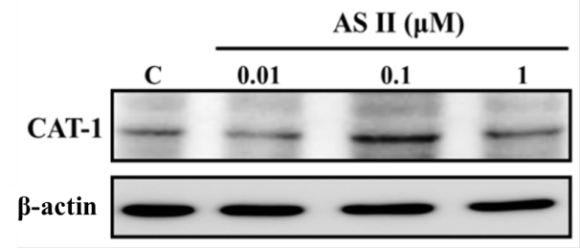


CAT-1

β-actin


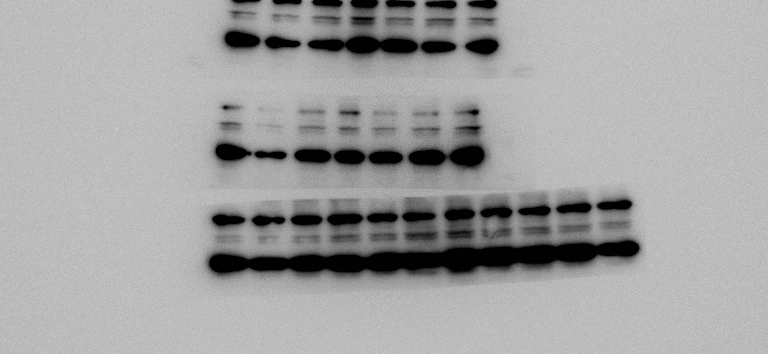

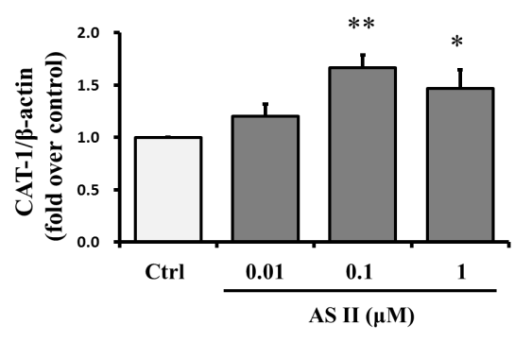


CAT-2


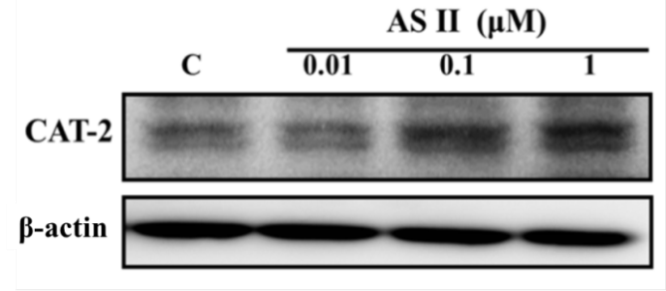


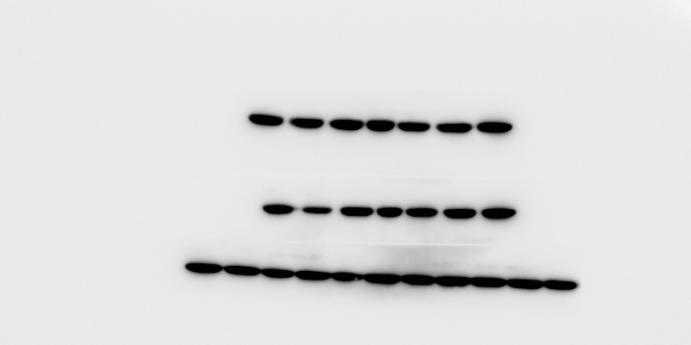

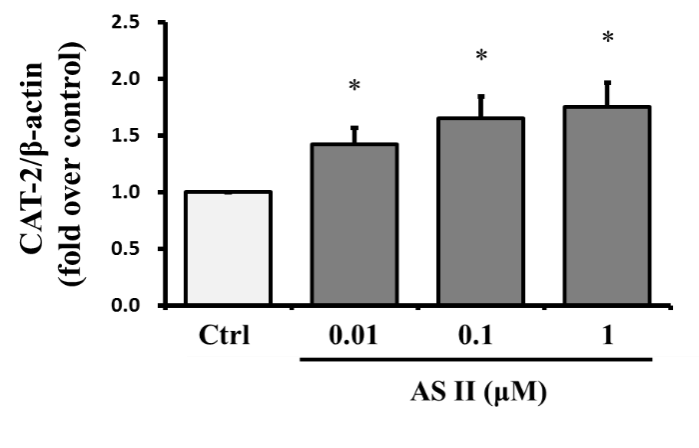


β-actin

β-actin

β-actin


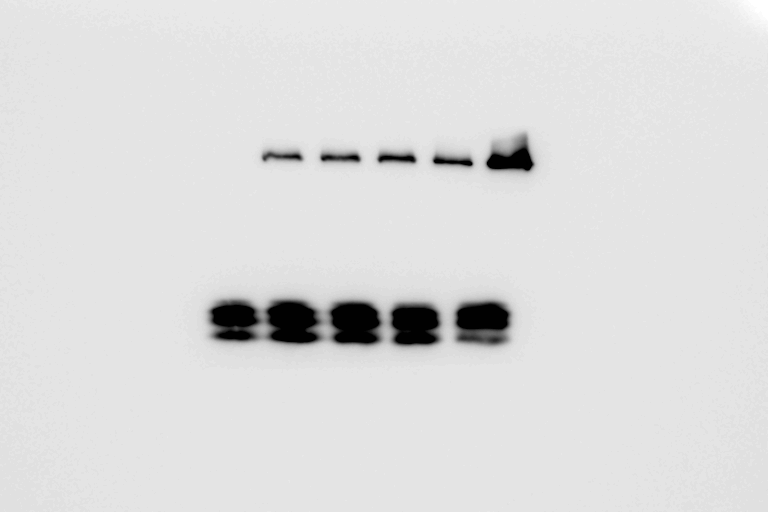


Figure 3

P-mTOR

(A)


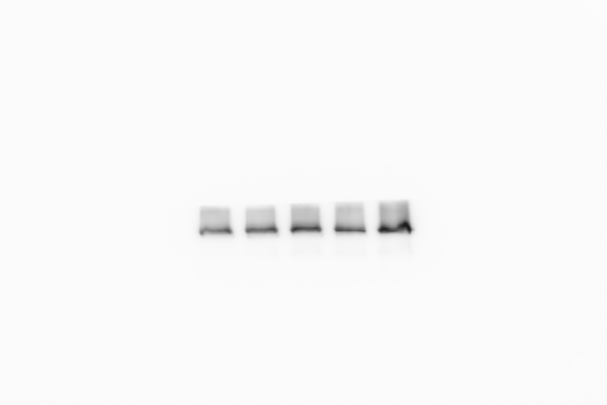

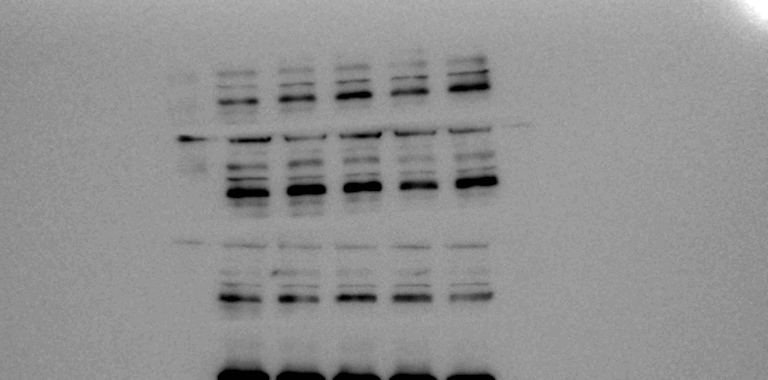

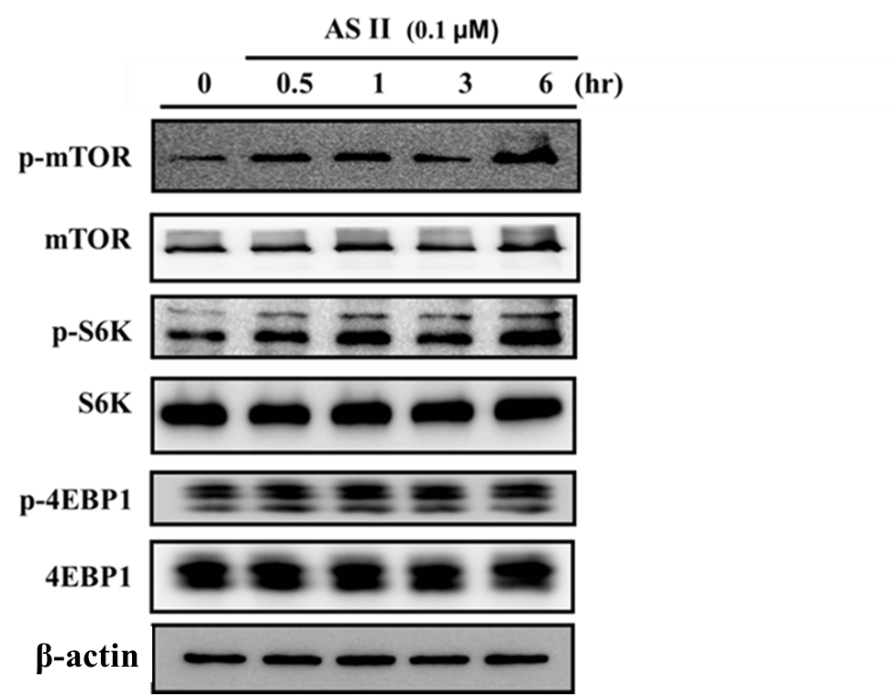


mTOR

p-S6K

(B)


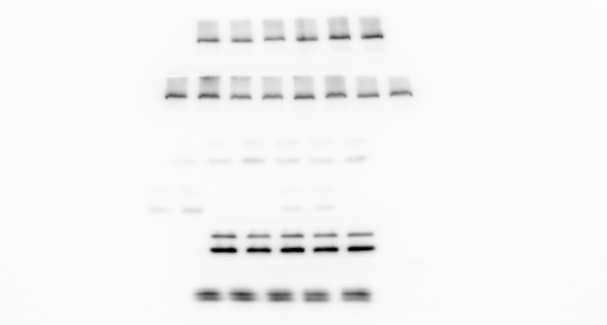

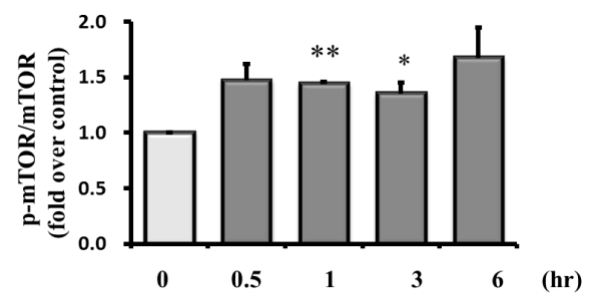


S6K


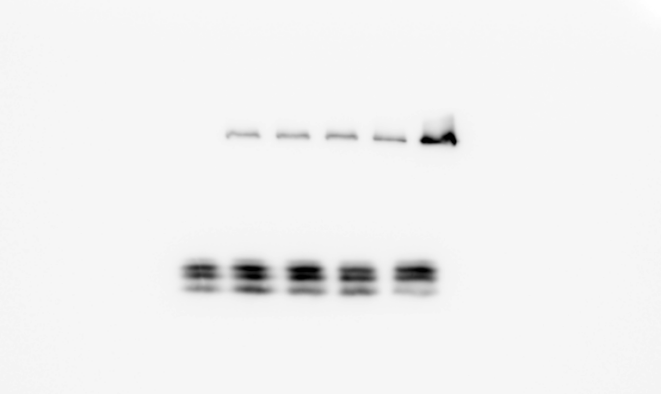
(C)


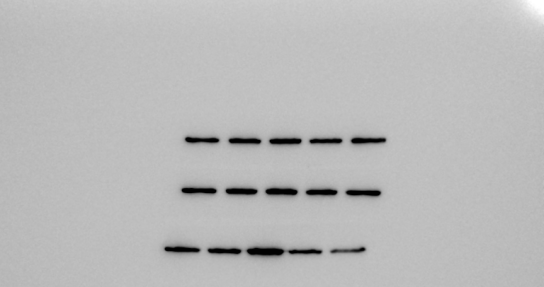

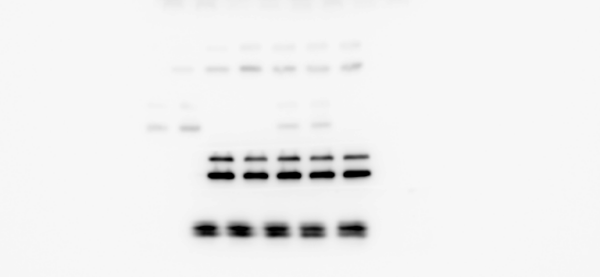

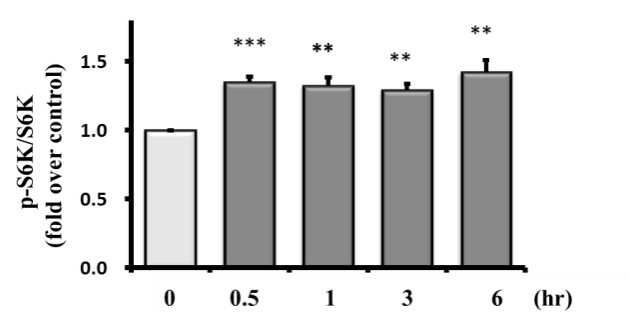


p-4EBP1

β-actin

4EBP1

(D)


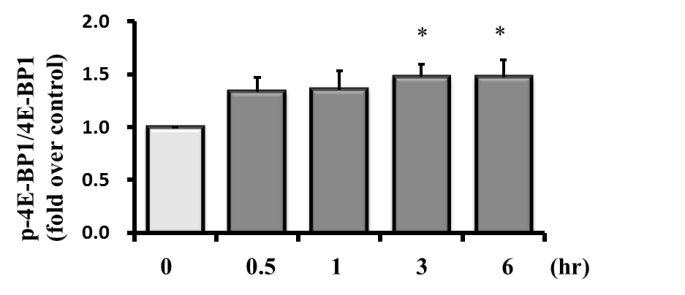


(E)


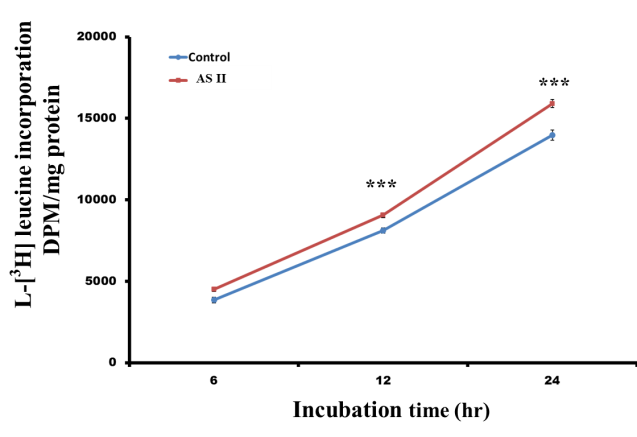


(F)


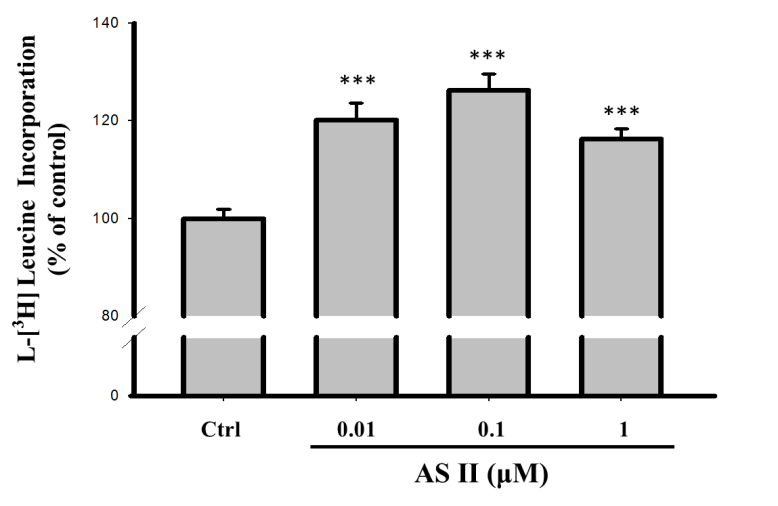


Figure 4

(A)


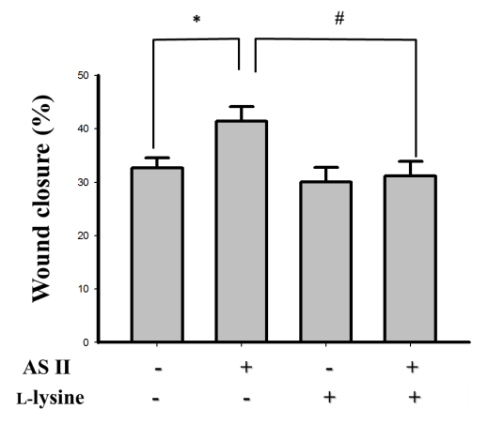


(B)x


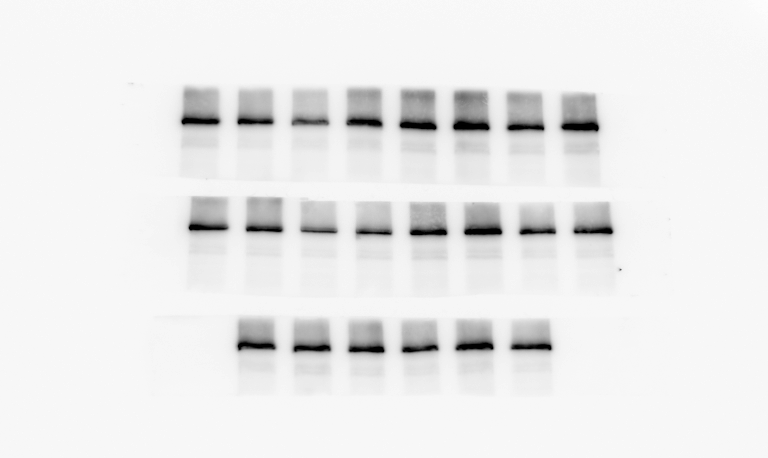

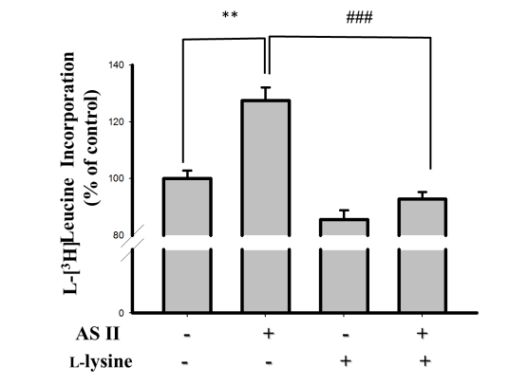


P-mTOR

(C)


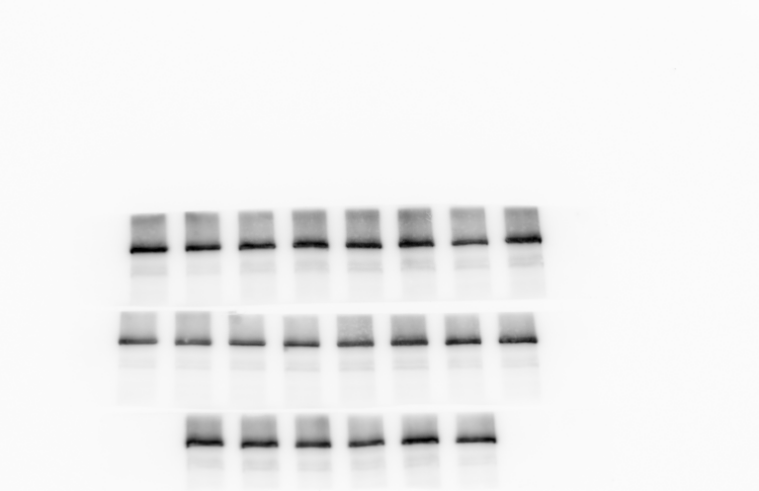

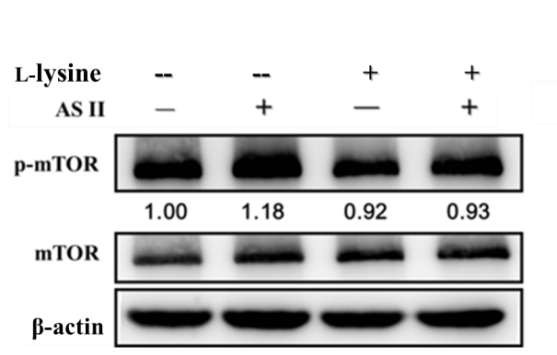


mTOR


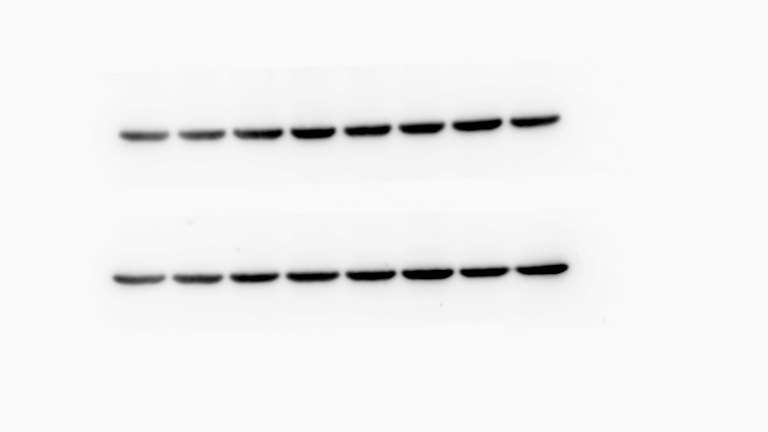
(D)

B-actin


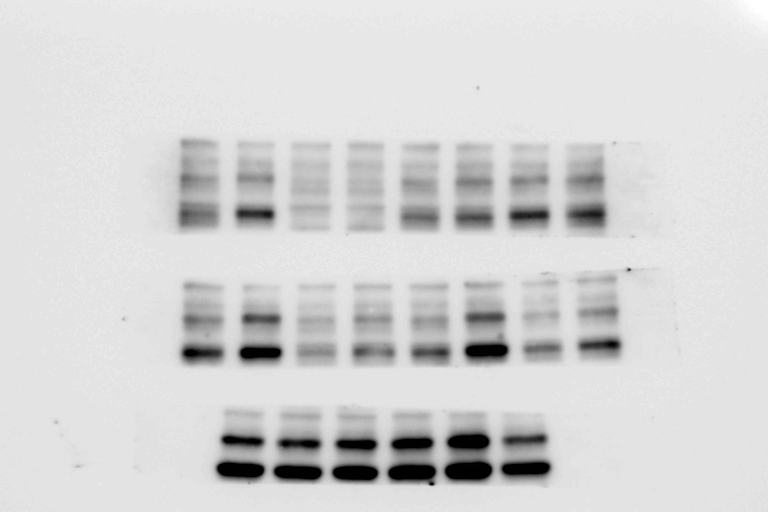

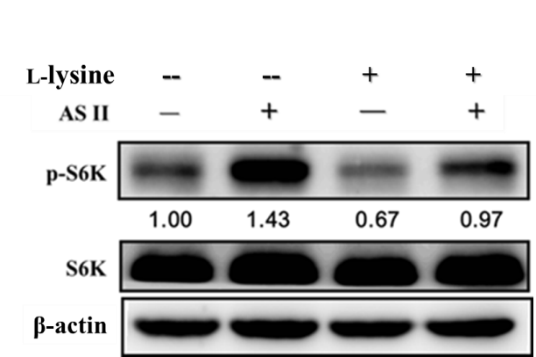


P-S6K


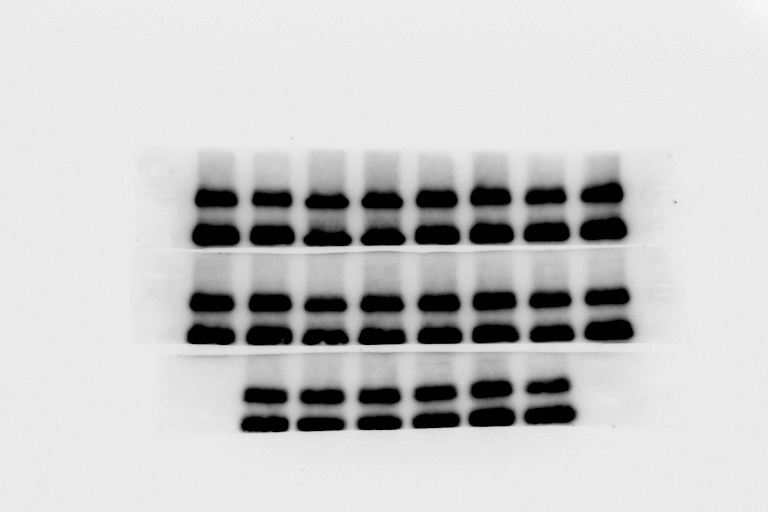


S6K

(E)


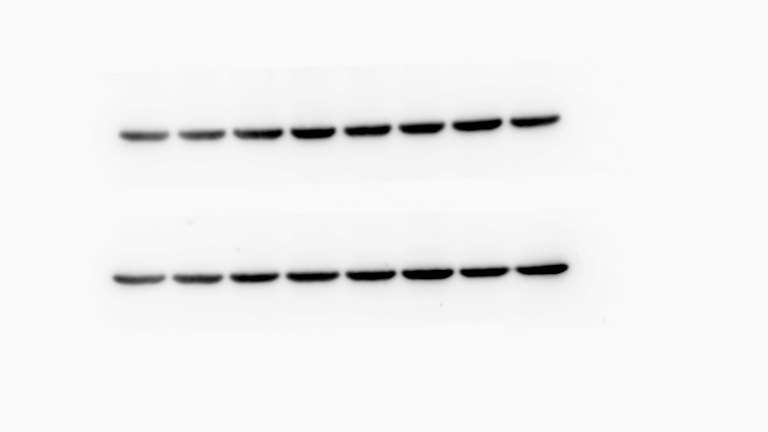

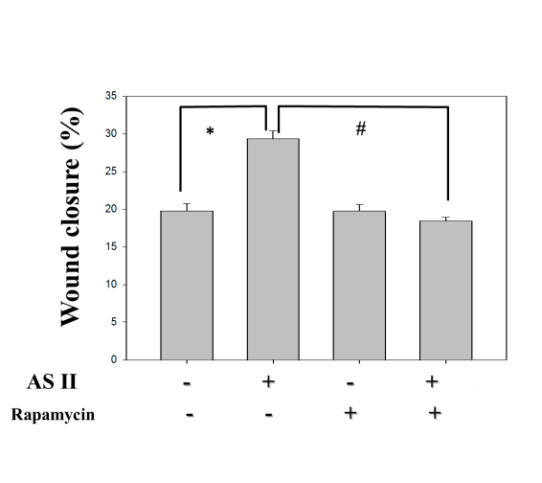


B-actin

(F)


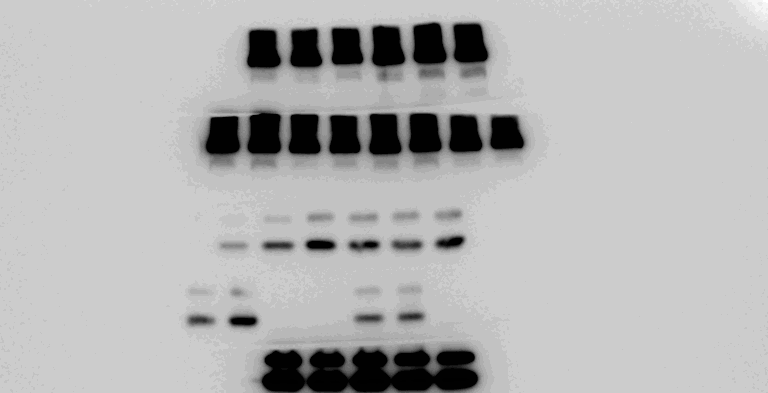

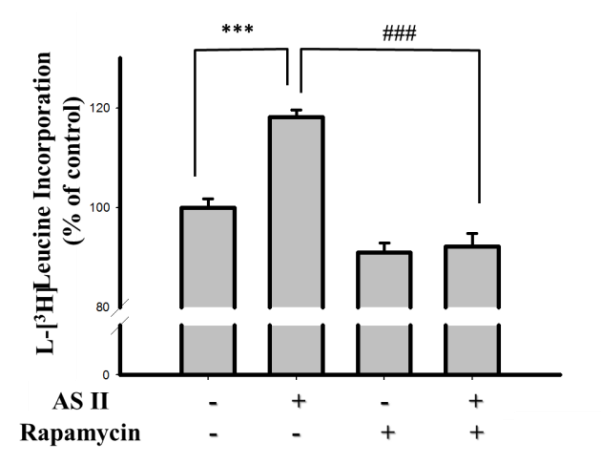


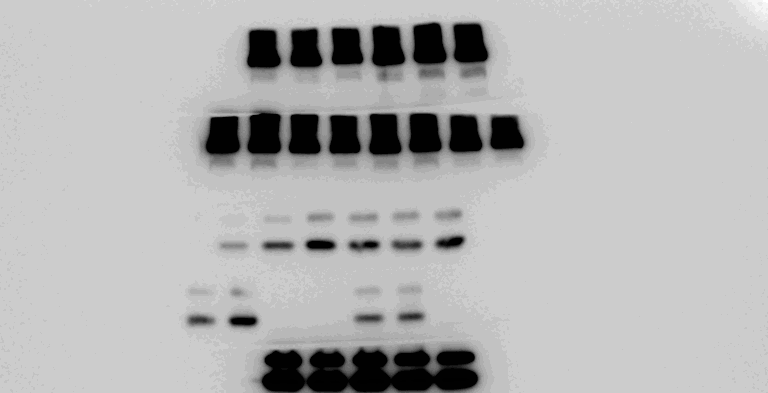


P-S6K

(G)


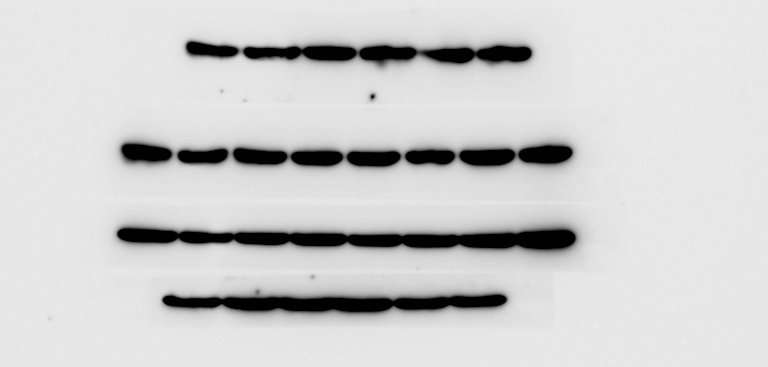

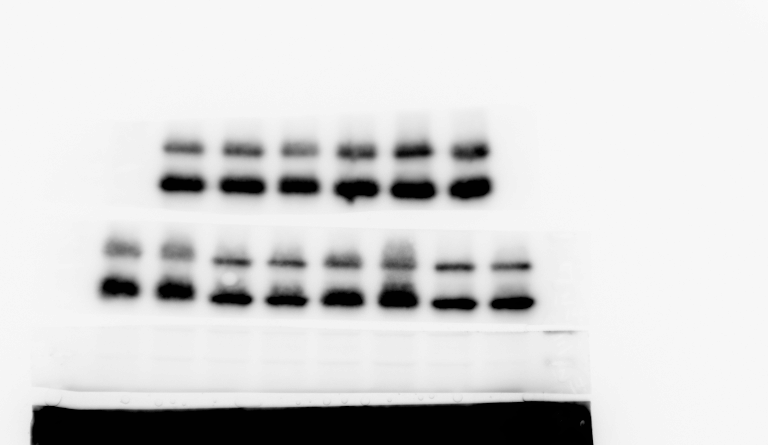

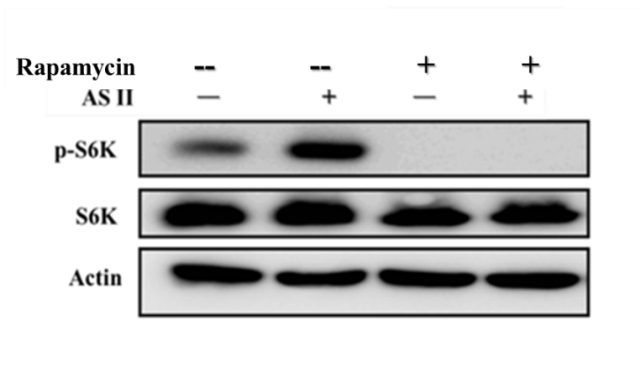


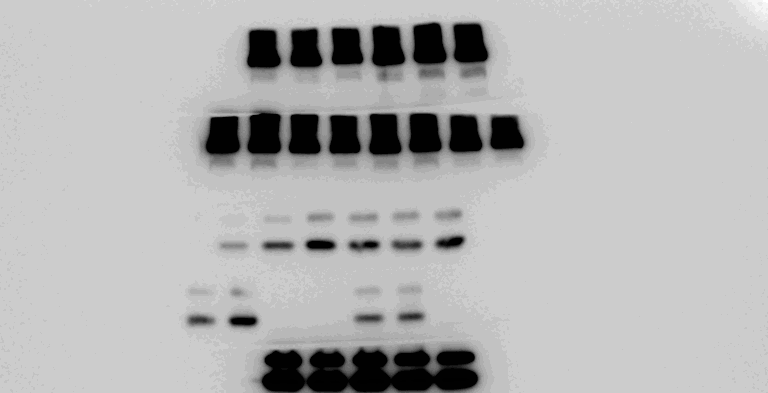


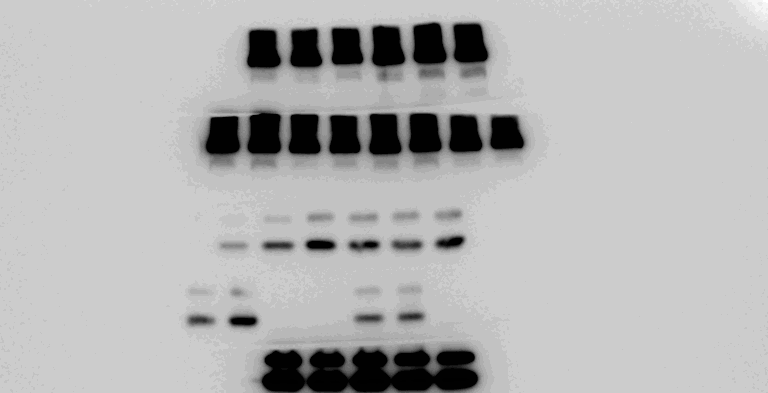


S6K


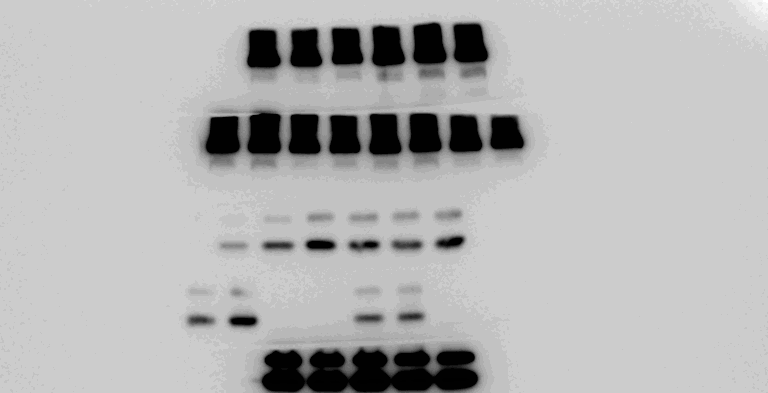


B-actin


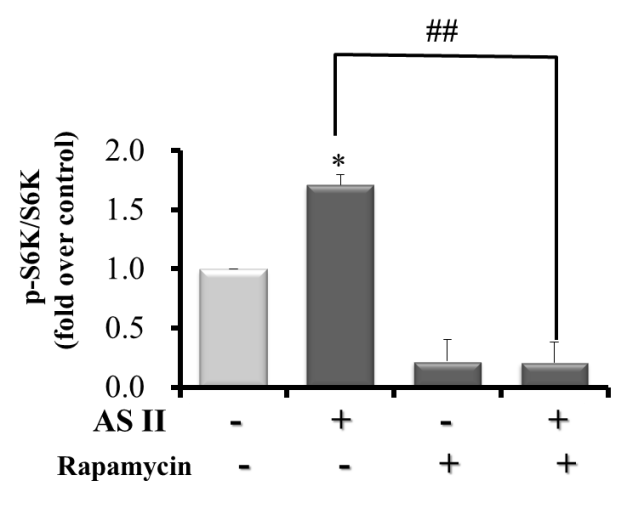


Figure 5

(A)


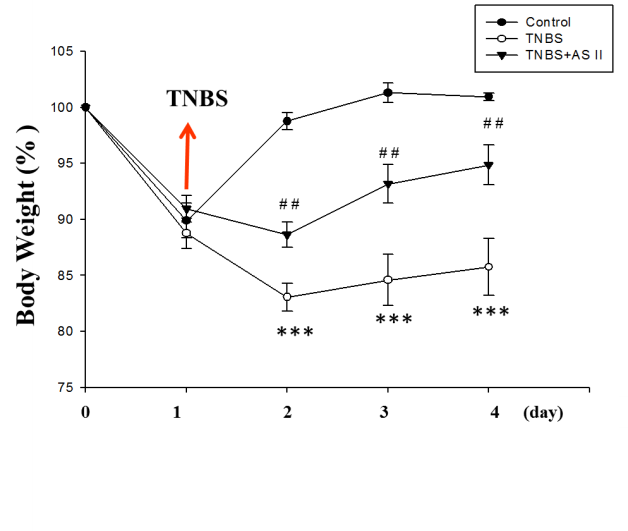


(B)


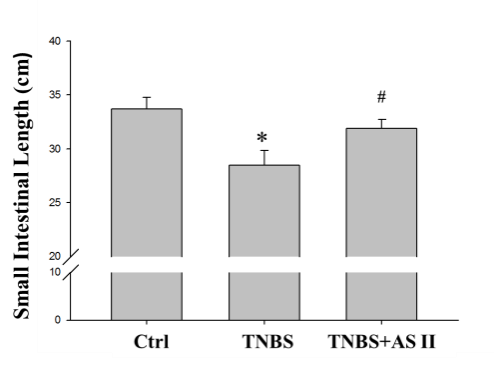


(C)


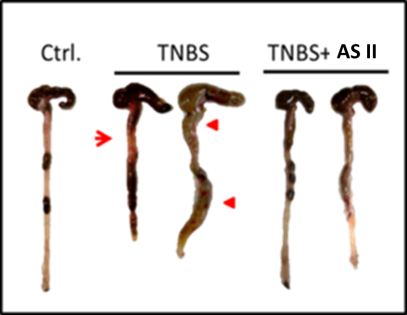


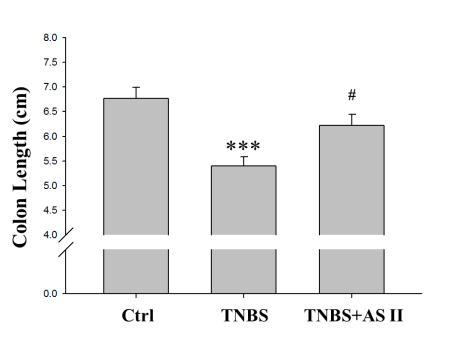


(D)


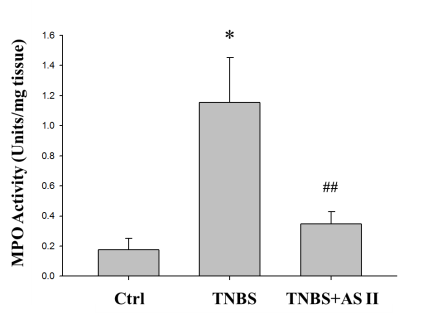


(E)


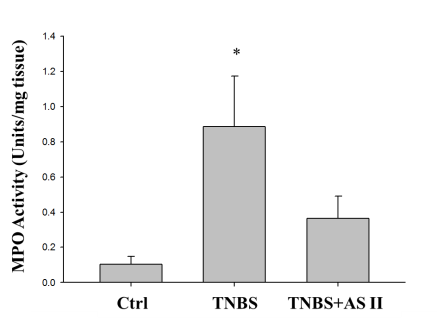


(F)


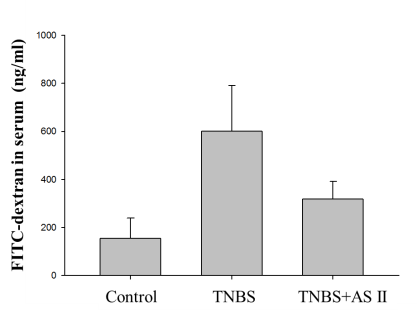


(G)


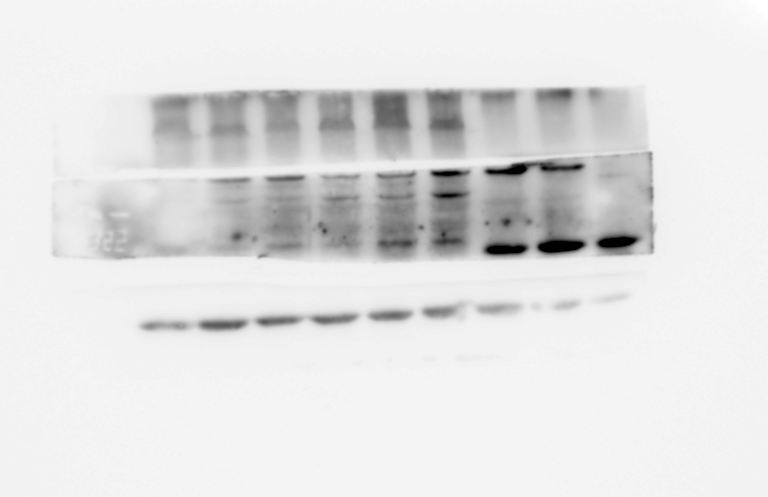

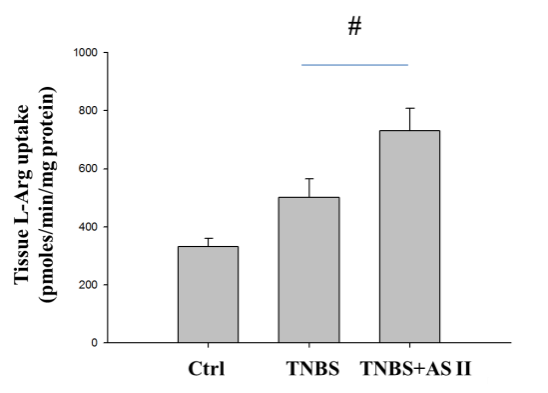


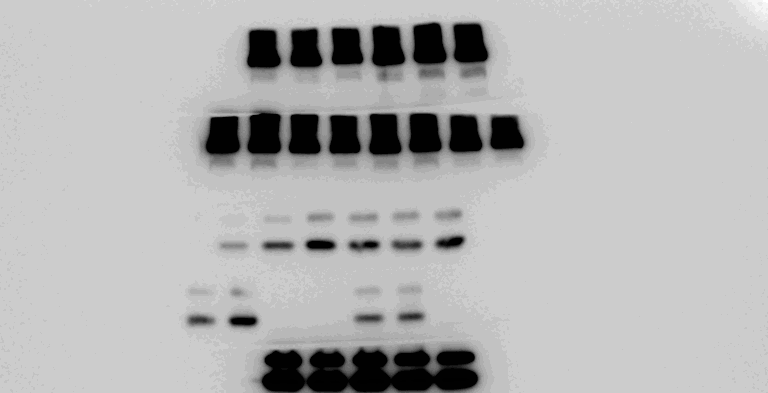


CAT-1


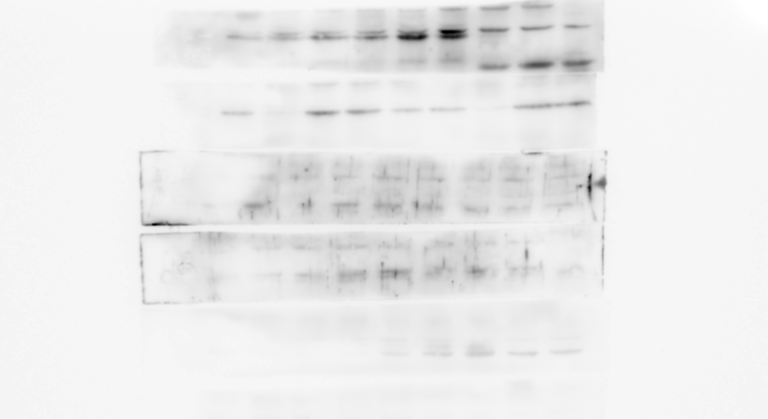


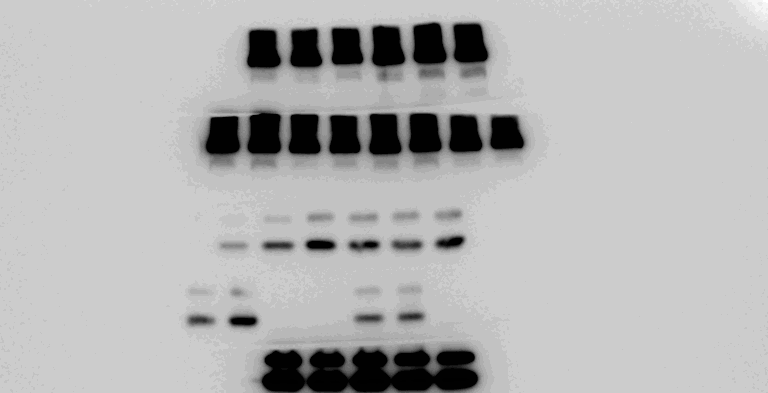


CAT-2

(H)


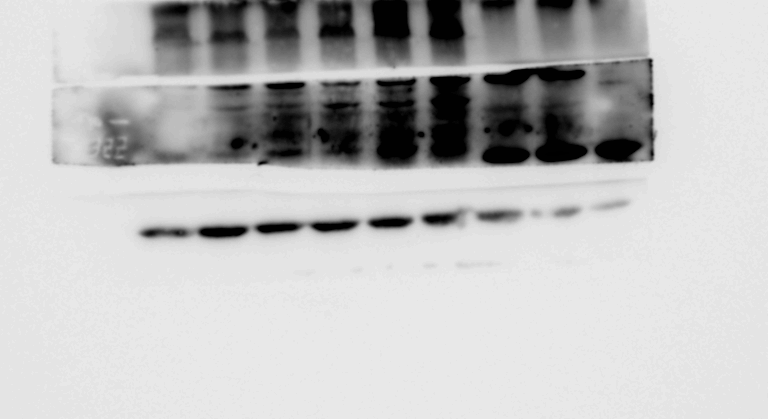

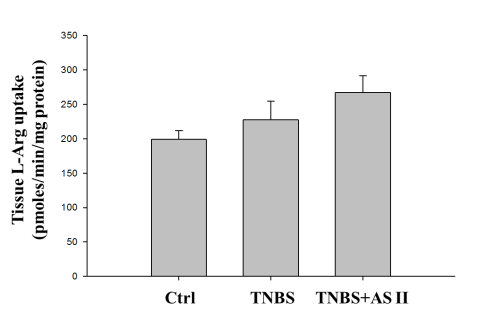


(I)


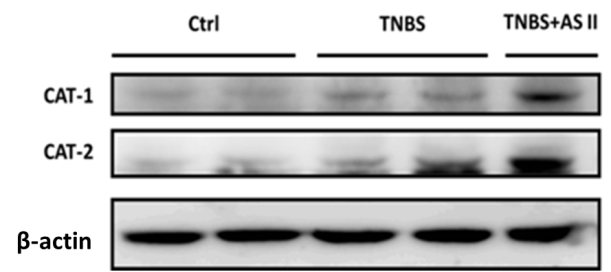


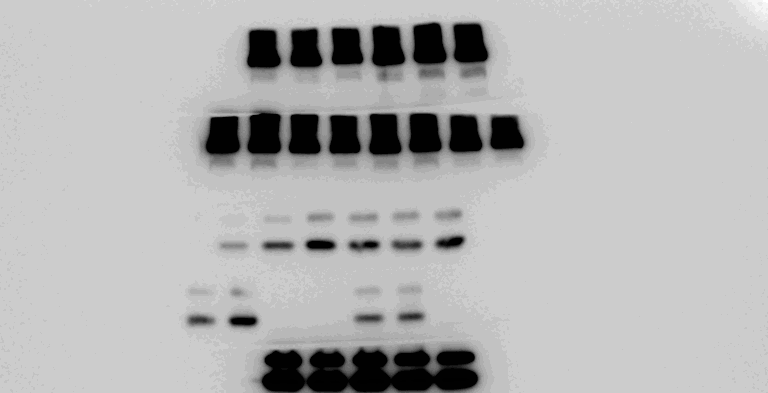


B-actin


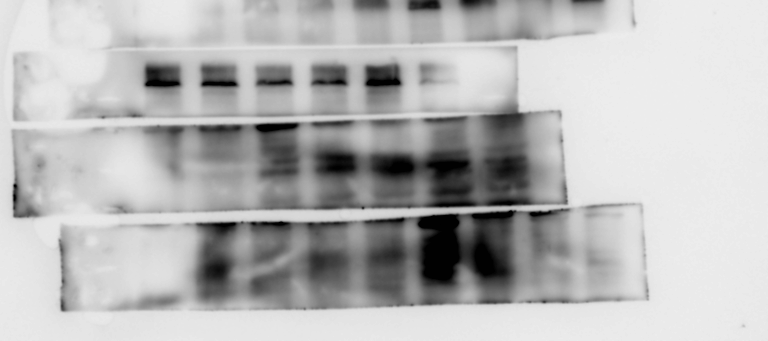
(J)


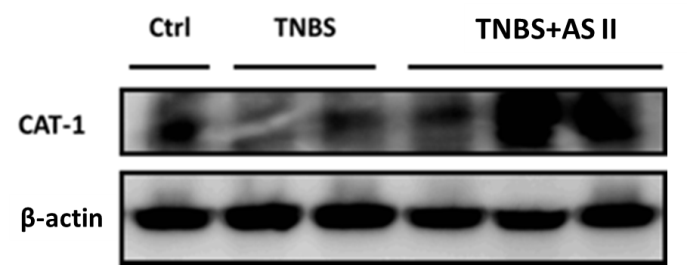


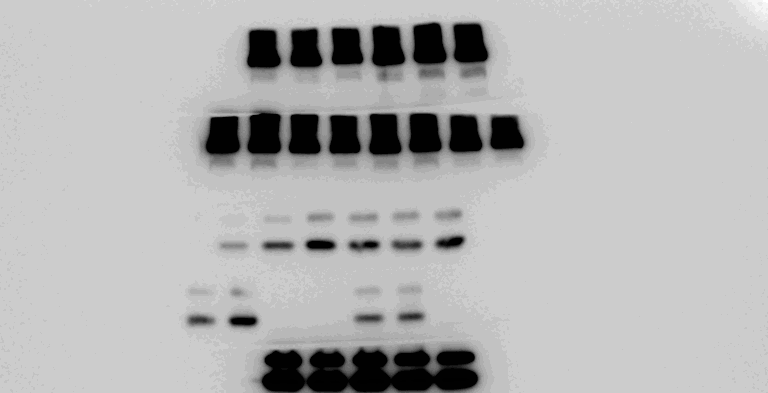


CAT-1


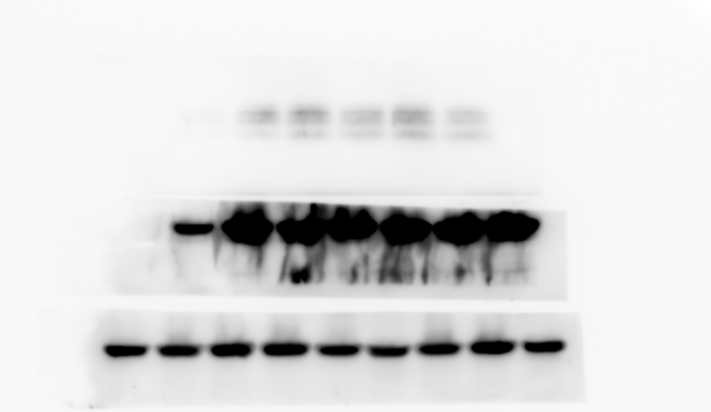


B-actin


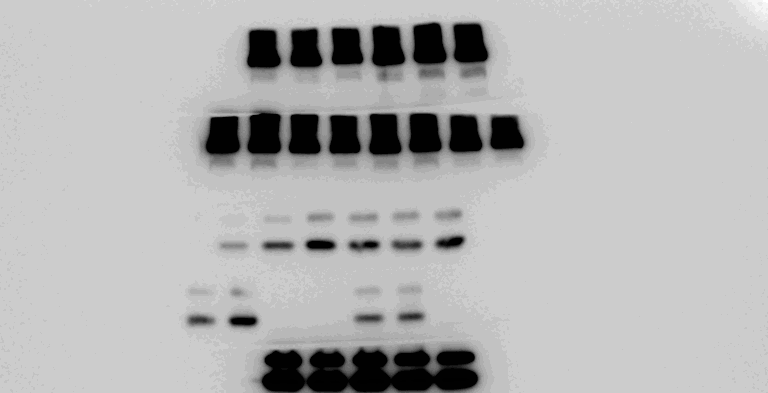


(K)


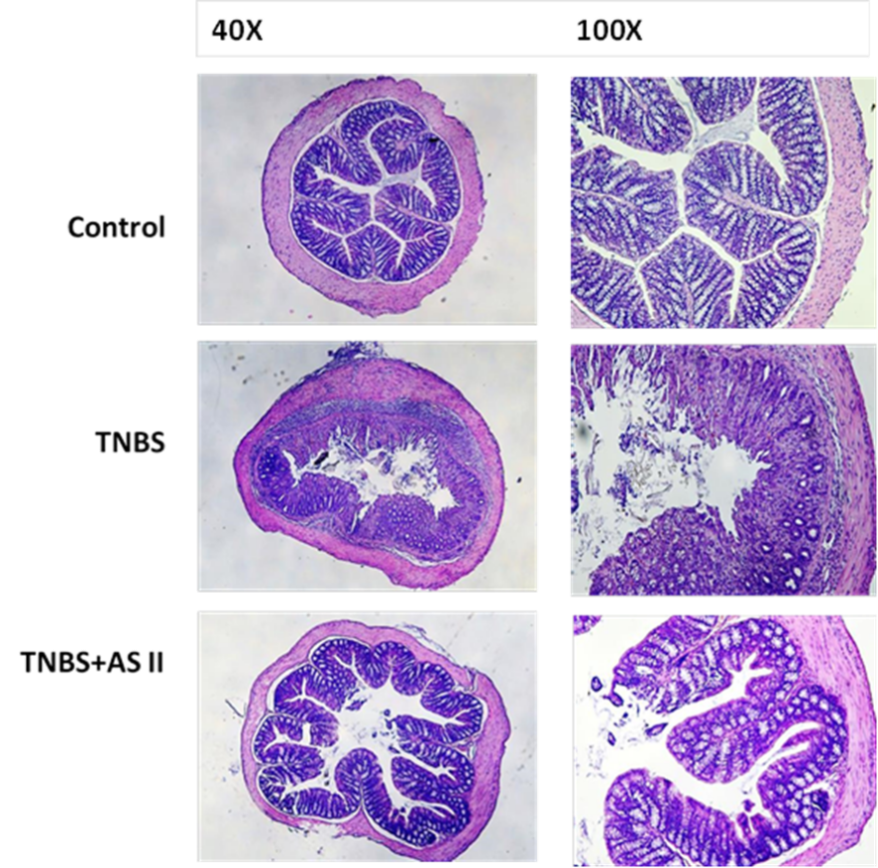


Figure 6


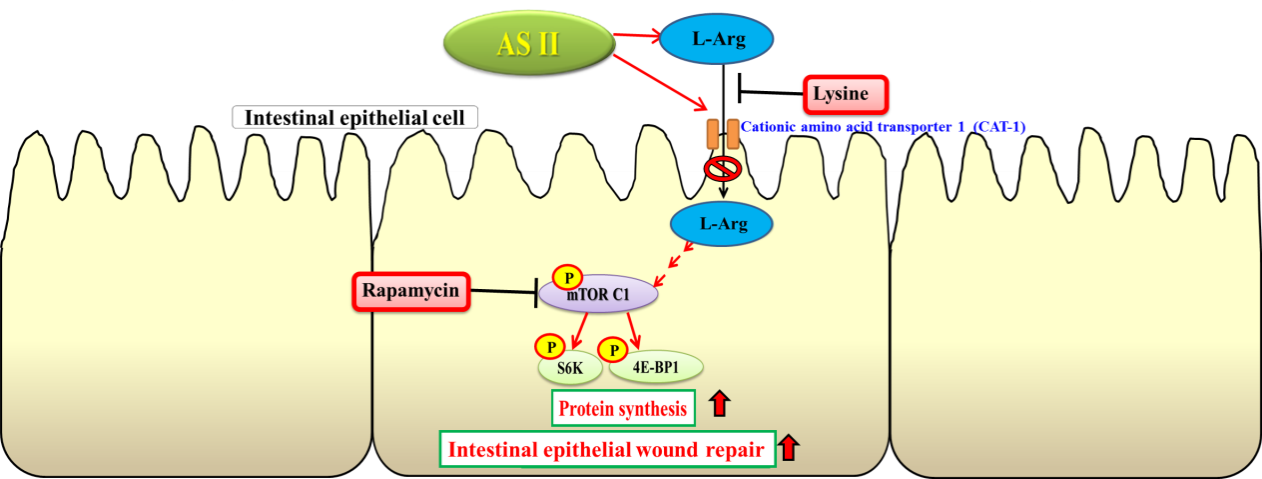

Supplement: Supplementary file 1 — Supplementary information [file 41598_2017_12435_MOESM1_ESM.doc]
